# Risk and Resilience Among Mothers and Fathers of Primary School Age Children With ASD in Malaysia: A Qualitative Constructive Grounded Theory Approach

**DOI:** 10.3389/fpsyg.2018.02275

**Published:** 2019-01-08

**Authors:** Kartini Ilias, Kim Cornish, Miriam Sang-Ah Park, Hasnah Toran, Karen Jennifer Golden

**Affiliations:** ^1^Jeffrey Cheah School of Medicine and Health Sciences, Global Asia in the 21st Century Research Platform (GA21), Monash University Malaysia, Subang Jaya, Malaysia; ^2^Department of Basic Sciences, Faculty of Health Sciences, Universiti Teknologi MARA, Puncak Alam, Malaysia; ^3^School of Psychological Sciences, Faculty of Medicine, Nursing and Health Sciences, Monash University, Melbourne, VIC, Australia; ^4^School of Social and Health Sciences, Leeds Trinity University, Leeds, United Kingdom; ^5^Faculty of Education, Universiti Kebangsaan Malaysia, Bangi, Malaysia

**Keywords:** resilience parenting stress, ASD, autism spectrum disorder, coping, Malaysia, qualitative, constructive grounded theory

## Abstract

Little is known about the coping and resilience experiences of parents of children with autism spectrum disorder (ASD) in the Malaysian cultural context. This study utilized a qualitative methodological approach adopting constructive grounded theory. The study sought to address the lack of research to date exploring the risk and protective experiences that contribute to parental stress and resilience for parents of primary school age children with ASD in the Malaysian setting. Twenty-two parents of children with ASD (13 mothers and 9 fathers) participated in semi-structured interviews. A strength of the study was the inclusion of both mother and father participant perspectives. The interviews lasted 50–80 min (mean: 67.5 min). The 22 parents had a total of 16 children (12 males; 4 females) formally diagnosed with ASD. Child age ranged between 5 and 12 years (mean age: 8.44). Overall, analysis of the 22 interviews revealed four prominent themes – *“initial reaction to child’s ASD symptoms and diagnosis,” “family life affected by a child with ASD,” “awareness about ASD in Malaysia,”* and *“coping strategies, wellbeing, and becoming resilient.*” The first three themes revolved around *stress and adversity*, and, the *adaptability* *and* *acceptance* of the parents. These processes illustrated the risks experienced by the parents of children with ASD in Malaysia. The last theme especially highlighted the *strengths and determination* of the parents and illustrated the protective experiences and processes that helped parents to develop and enhance resilience. Overall, the findings revealed that resilience develops synergistically and dynamically from both risk and protective experiences across different levels – individual, family, community, society and government. The findings motivated the development of our theoretical model of resilience that can help health and education professionals tailor assessment and interventions for parents of children with ASD in the Malaysian context. Clinical, policy, and research suggestions were discussed.

## Introduction

There is ample evidence that raising a child with autism spectrum disorder (ASD) is challenging and complex (Gray, 2002a; Meirsschaut et al., 2010; Weiss et al., 2012; Hayes and Watson, 2013; Cridland et al., 2014; Ooi et al., 2016). The clinical diagnostic symptoms of ASD include (a) difficulties in social communication and social interaction across various contexts, as well as (b) an inclination to engage in restricted, repetitive patterns of behavior, interests, or activities (American Psychiatric Association, 2013). Findings from previous studies have shown that the presence of children with ASD in the family impacts various domains of parental and family life (Hayes and Watson, 2013), such as the marital relationship, sibling relationships, family socialization patterns and family routines, etc. The psychosocial aspect of wellbeing in parents and families of children with ASD warrants specific attention in research (Lecavalier et al., 2006; Meadan et al., 2010), as a child with ASD needs greater understanding from the parents, extended family, friends, the community, and professional caregivers (e.g., Quintero and McIntyre, 2010). ASD has been the subject of a large and growing area of research in Western countries (Herring et al., 2006; Firth and Dryer, 2013; McStay et al., 2014). However, problematically, there is scant research that explores these subject matters in non-Western countries, which includes Malaysia (Samadi and McConkey, 2011; Daley et al., 2013; Freeth et al., 2014; Neik et al., 2014; Ilias et al., 2018).

In Malaysia, there have been increasing reports from doctors, psychologists, and psychiatrists with regards to the increasing number of children with ASD referred to their clinics (Toran, 2011; Ministry of Health Malaysia, 2014; Neik et al., 2014). Moreover, the number of children enrolled in special needs programs has also reportedly doubled between 2006 and 2013 (Toran, 2011; Razali et al., 2013). Together, these findings suggest the need for more autism-related research in Malaysia. It is, however, most likely that the response of parents and families to a child’s ASD diagnosis would vary from family to family. Showcasing this coping variability is the growing interest in research to discover why and how some parents and families of children with ASD manage to function well and become stronger, whereas, others do not cope so well (e.g., Walsh, 1998; Patterson, 2002; Bekhet et al., 2012; Bayat and Schuntermann, 2013; Leone et al., 2016).

This area of inquiry has in turn given rise to the development of a field known as *family resilience* and efforts to understand resilience in a relational context. Walsh’s (1998, 2003, 2016) *Family Resilience Framework* has been widely adopted as one of the key theoretical frameworks in working with families facing adversity and stressors. Walsh (2003) articulated a theory of family resilience, drawing together findings from studies of individual resilience as well as research on effective family functioning. Her framework identifies a number of domains and qualities of family members that contribute to resilience: *family belief systems* (such as spirituality, maintaining a positive outlook, etc.), *family organizational processes* (interpersonal relationships, effective social networks, and economic resources) and *family communication processes* (collaborative communication and problem solving).

Walsh’s family resilience theory aims to investigate the factors that contribute to healthy family functioning rather than family deficits (Hawley and DeHaan, 1996). The concept of family resilience extends our understanding of normal family functioning to situations of adversity (Walsh, 2016). This concept is applicable in understanding how parents and family members of children with ASD function as a unit when facing the challenges, difficulties and hardships of raising a child with ASD. Specific to the current paper, families with adversity and stressors would refer to families with a child (or children) diagnosed with ASD.

Resilience has been defined in various ways in the past literature. Recent writings are increasingly highlighting the nature of resilience as a *dynamic process* (e.g., Stainton et al., 2018), as resilience involves the dynamic interaction between perceived risk and a range of protective, attenuating, and recovery factors that can help a person to adapt well in the face of adversities and to thrive. Resilience can be understood as a dynamic process made up of many things, including but not limited to adaptive coping behaviors. Resilience resources can be sourced from personal, relational, and environmental life domains. A similar, but still distinct construct from resilience, *coping* can be viewed as involving purposeful attempts to manage stress, regardless of the effectiveness (Compas et al., 1988). Lazarus and Folkman (1984) classically defined adaptive coping as referring to cognitive and behavioral efforts to manage internal and external demands that are taxing or exceeding the person’s resources to cope.

Despite the emerging body of literature on the psychosocial wellbeing and resilience of parents of children with ASD (Bekhet et al., 2012; McConnell et al., 2014; Leone et al., 2016), the risk and protective processes have yet to be explored extensively in a collectivistic culture such as Malaysia (Keshavarz and Baharudin, 2009; Ting and Chuah, 2010; Ravindran and Myers, 2012). Given the fact that traditions, beliefs and values are parts of the cultural piece that could influence and shape how the families of a child with ASD perceive their life experiences and challenges of raising a child with ASD, these factors (cultural factors) are likely to play a role in coping and resilience (Ghosh and Magana, 2009; Cridland et al., 2014; Xue et al., 2014). People who live in a collectivistic culture tend to be more interdependent on each other (Kitayama et al., 2009; Selin, 2013). Their emphasis on in-group relationships and harmony may also influence the way they make sense of experiences and challenges, influencing their coping behaviors as well as their use of the resources available (Dyches et al., 2004; Lam and Zane, 2004). The meanings of health, illness, and disability in regard to ASD can vary a great deal across cultures and across time (Ravindran and Myers, 2012). For example, within the South-East Asia regional context, both cultural similarities and cultural differences have been identified in a systematic literature review on parenting stress and resilience when parenting a child with ASD (Ilias et al., 2018).

Thus, the current study seeks to address the lack of research to date exploring the experiences, risks and protective processes that contribute to parental stress and resilience for parents of children with ASD in the Malaysian setting. Furthermore, there has been little research that explored qualitatively the experiences, parenting stress and resilience of the parents (Ting and Chuah, 2010; Ilias et al., 2017). Thus, the qualitative nature of this study allows for a deeper analysis, while allowing rich cultural data to be shared in the area where scarce information is present.

Previous studies on risk and resilience in parents of children with ASD have often focused on mothers as opposed to fathers; thus, there remains a gap of research regarding fathers of children with ASD and their experiences (Allen et al., 2013; Braunstein et al., 2013). Also, the experiences of mothers and fathers of children with ASD have been found to differ (e.g., Falk et al., 2014). Mothers were often found to play the primary caregiver role (e.g., Braunstein et al., 2013) and this role expectation may be especially strong in more traditional societies. Additionally, Jones et al. (2013) identified higher levels of psychological distress (stress, anxiety, and depression) in mothers compared to fathers, and also higher levels of reported positive gains in mothers compared to fathers. Parenting stress may be experienced differently by mothers and fathers of children with ASD and may depend on multiple factors; the research remains unclear about the effect of parent gender on risk and resilience (Hayes and Watson, 2013).

No formal hypotheses were formulated as the study was exploratory and used a constructive grounded theory approach (Charmaz, 2006; Creswell, 2007). Well-documented evidence suggests the presence of stressors and challenges in families of children with ASD; yet, past research has highlighted that most parents and families of children with ASD were able to rise above challenges (Gray, 2002b; Woodgate et al., 2008; Bekhet et al., 2012). Thus, it was anticipated that parents of children with ASD would highlight *both* positive (growth) experiences as well as challenging (stress) experiences of raising their child with ASD. Similarly, it was anticipated that culturally specific experiences and processes would be shared by participants that would differ from the themes mentioned in previous literature in Western countries (e.g., Ooi et al., 2016). Unique culturally specific themes might relate to a stronger emphasis on the lack of resources available in the Malaysian context and associated financial challenges; the stigma present in Malaysia; as well as the role of spiritual supports (Ilias et al., 2017).

## Materials and Methods

### Participants

Of the 22 parents of a child with ASD who volunteered to participate in the interview, 13 were mothers and 9 were fathers. Six parents were interviewed in pairs (3 fathers and 3 mothers). Sixteen parents were interviewed individually. To clarify, 3 fathers were interviewed together with their wives; whereas, 6 fathers were interviewed alone. The parents’ ages ranged from 32 to 50 years. In terms of their ethnic backgrounds, 19 of the parents identified as Malay, one as Chinese, and two as Indian. Seventeen of the parents resided in the Klang Valley, an urban region, including the capital city Kuala Lumpur and surrounding areas. Two parents were living in Negeri Sembilan, which lies on the Western coast of peninsular Malaysia just south of Kuala Lumpur and borders Selangor on the south. Three parents were living in Melaka, which is in the southern region of peninsular Malaysia. Two parents, a mother and a father, were from Sabah, East Malaysia; however, they were residing currently in Klang Valley. Relationship to the child, religion, ethnicity, marital status, education level, and employment status are illustrated in Table 1.

**Table 1:** Demographics of mothers and fathers of children with ASD.

Number	Participant	Relationship to child	Age Range	Race/ethnicity	Religion	Marital status	Level of educatio	Employment status
1	Aneesa	Mother	40–50	Malay	Muslim	Married	Degree	Free Lance
2	Ahmad	Father	40–50	Malay	Muslim	Married	Degree	Free Lance
3	Beetha	Mother	30–40	Indian	Christian	Married	Degree	Med Doctor
4	Camilah	Mother	30–40	Malay	Muslim	Married	Degree	Housewife
5	Delina	Mother	30–40	Malay	Muslim	Married	Degree	Teacher
6	Farena	Mother	30–40	Malay	Muslim	Married	Degree	Executive
7	Ghaida	Mother	40–50	Malay	Muslim	Married	High school	Housewife
8	Farid	Father	40–50	Malay	Muslim	Married	High school	Clerk
9	Hana	Mother	30–40	Malay	Muslim	Married	PhD	Lecturer
10	Imran	Father	30–40	Malay	Muslim	Married	PhD	Lecturer
11	Junaina	Mother	30–40	Malay	Muslim	Married	Degree	Housewife
								Previously was a teacher
12	Keen	Mother	40–50	Chinese	Free Thinker	Married	Degree	Housewife
								Previously was an executive
13	Leena	Mother	30–40	Indian	Christian	Married	Diploma	Housewife
14	Liya (Intan)	Mother	30–40	Malay	Muslim	Married	Degree	Housewife
								Previously was an executive
15	Mawi	Father	30–40	Malay	Muslim	Married	Diploma	Executive
16	Nasyran	Father	30–40	Malay	Muslim	Married	Diploma	Government Servant
	(Jo)							
17	Tahera	Mother	30–40	Malay	Muslim	Married	Degree	Executive
18	Razila	Mother	30–40	Malay	Muslim	Married	Degree	Senior IT executive
19	Ramli	Father	30–40	Malay	Muslim	Married	Master	Senior IT engineer
20	Rashid	Father	30–40	Malay	Muslim	Married	Degree	Research officer
21	Suhaimi	Father	30–40	Malay	Muslim	Married	Master	Lecturer
22	Tahir	Father	40–50	Malay	Muslim	Married	Master	Lecturer


*All names have been changed to pseudonyms; parents’ individual ages are reported in 10-year intervals to protect confidentiality.*

The 22 parents had a total of 16 children (12 males; 4 females) formally diagnosed with ASD (Table 2). Child age ranged between 5 and 12 years (mean age: 8.44). Previous studies have suggested that parents may experience stress, cope, and perceive parenting self-efficacy differently depending on the child’s developmental stage (Mash and Johnston, 1983; Gray, 2002a; Tobing and Glenwick, 2002; Kuhn and Carter, 2006). Thus, it was judged as important to target one child age group for the current study to ease the achievement of theoretical data saturation and to develop a focused theoretical model. Also, before the present study began, exploratory pilot interviews were conducted with 11 parents of children with ASD using Interpretative Phenomenological Analysis (IPA) qualitative methods, and it was observed the parents of primary school age children presented with different experiences than the parents of adolescents and adults. This observation helped guide the researchers to narrow down the age group and specify the inclusion criteria to include parents of a primary school aged child diagnosed with ASD. The age requirement (5–12 years) also enabled matching of this qualitative sample with a related quantitative study by the authors, which was part of a bigger mixed methods project.

**Table 2:** Details of child with ASD for each parent(s).

Parent (s)	Child	Child’s age and gender	Age diagnosed	Number of people in household	Child school type
Aneesa and Ahmad	Amni	12, female	5 years 8 months	4	Community Rehabilitation Centre supported by government in the morning (4 h). Primary education in typical government school.
Beetha	Bevan	6, male	2 years 8 months	6	Private special education school and private therapies.
Camilah	Danish	9, male	6 years 4 months	5	Community Rehabilitation Centre supported by government in the morning (4 h). Primary education in local government school in special education class.
Delina and Suhaimi	Emran	7, male	4 years 11 months	5	Community Rehabilitation Centre supported by government in the morning (4 h). Primary education in local government school in special education class.
Farena	Faizul	7, male	6 years 5 months	7	Primary education in local government school and private therapies.
Ghaida and Farid	Ghazali	10, male	5 years 8 months	6	Community Rehabilitation Centre supported by government in the morning (4 h). Primary education in local government school in special education class.
Hana and Imran	Hamdi	8, male	6 years 4 months	5	Private religious school and therapies at non-profit autism services centre.
Junaina	Jameela	9, female	4 years 4 months	5	Primary education in local government school in special education class. Therapy at non-profit autism services centre.
Keen	Kevin	9, male	3 years 8 moths	4	Private mainstream school and private non-profit therapy
Leena	Leo	8, male	2 years 7 months	4	Primary education in local government school.
Leeya and Mawi	Laili	7, female	4 years 10 months	5	Primary education in local government school in special education class and continue private therapy.
Nasyran	Nabila	8, female	4 years 6 months	6	Primary education in local government school in special education class.
Taheera	Tamim	7, male	3 years 10 months	5	Primary education in local government school in special education class.
Razila and Ramli	Rafiq	9, male	2 years 6 months	5	Primary education in local government school in special education class. Continue therapy and enhancement class at a government autism early intervention program.
Rashid	Syed	8, male	6 years 4 months	5	Primary education in local government school and continue therapy and enhancement class at a government autism early intervention program.
Tahir	Tamir	11, male	3 years	7	Community Rehabilitation Centre supported by government in the morning and afternoon, Primary education in local government school in special education class.


*All names (parents and children) have been changed to pseudonyms. The age of diagnosis reported in the time intervals was identified by the parents themselves (i.e., months or years).*

The target age of primary school age children was also chosen as there is evidence that the younger the implementation of mental health promotion and resilience programs, the greater the positive effect (Fenwick-Smith et al., 2018). Thus, we hoped to gather the risk and resilience experiences from these participants in order to inform development of early intervention support prevention programs. Focusing on the pre-school period would have been difficult, especially in this region, given the late (delayed) diagnosis of children. Moreover, compared to the pre-school period, focusing on the primary-school age period would allow the parents to reflect on the developmental trajectory of developing resilience. The study also plans to have a longitudinal follow-up, so re-interviews will occur later in adolescence and early adulthood, thus, the starting point at primary school age was selected. Also, primary school age is a crucial transition period that often places high demands on the parents and children with ASD (Stoner et al., 2007; Fontil and Petrakos, 2015) and we hoped the processes of both risk and resilience would be elucidated.

It has been argued that there is no one size fits all method to reach data saturation due to the variability of research designs (Fusch and Ness, 2015). Charmaz (2006) described that there remains disagreement about the definition of data saturation. There are a few guidelines that have been proposed in regards to theoretical data saturation. Creswell (1998) recommended that 20–30 interviews may constitute sufficient data for a grounded theory study. Charmaz (2006) highlighted that within grounded theory methodology the focus is on “theoretical saturation”; data saturation does not mean stopping gathering new data when a repetitive pattern occurs, but to continue conceptualization of comparisons of stories and incidents to yield different properties of the pattern until no new properties of the pattern emerge.

### Design

Grounded Theory, developed in 1967 by Glaser and Strause, is defined as “the discovery of theory from data systematically obtained from social research” (p. 2). Grounded theory is a qualitative research methodology (Creswell, 1998). Moreover, there are several types of grounded theory such as Staussian Grounded Theory, Glaserian Grounded Theory, dimensional analysis, constructivist, and situational analysis. Commonalities include the use of relatively unstructured interviews, use of specific techniques to categorize data and identify characteristics, and the interest in interactions and processes (Morse and Niehaus, 2009).

This study adopted Charmaz’s (2006) social constructivist perspective of grounded theory. Charmaz’s social constructivist perspective “emphasizes diverse local worlds, multiple realities and the complexities of particular worlds, views and actions” [p. 65] and recognizes that “the ‘discovered’ reality arises from the interactive process and its temporal, cultural, and structural contexts” (Charmaz, 2000, p. 524). In-depth, flexible, semi-structured interviews were used to capture the participants’ perspectives and life experiences. This method was chosen due to the complexities of the reality of life experiences of the parents raising a child with ASD. Charmaz views grounded theory methods “as a set of principles and practices, not as prescriptions or packages” (Charmaz, 2006, p. 9) and emphasizes “flexible guidelines, not methodological rules, recipes and requirements” (Charmaz, 2006, p. 9). The constructive grounded theory approach assumes that data and theories are neither emergent nor discovered, but rather are constructed by both the researcher and the research participant (Charmaz, 2006; Allen, 2010).

Parent interviewees did not express a desire to review the interview transcripts secondary to time constraints (their very busy schedules), although this possibility was presented as an opportunity for them. In this study, an additional interview with a mother of a teenager with ASD, who also works as an applied behavior analysis (ABA) consultant was used as a method of *data triangulation*, which helped to increase the credibility and trustworthiness of the data. Key informant interviews can be a useful strategy for gaining information from “experts” who have been in a position to put their own interpretations into practice and can share their experiential knowledge (Bogner et al., 2009). She experienced raising her child in the Malaysian context and has worked to empower parents in the country. She reviewed the thematic analysis findings during an audio-recorded interview of approximately 40 min, and she described that the thematic structure conveyed a very good understanding of the Malaysian situation according to her personal and professional experience.

During the last phase of the study, community engagement events were conducted. This involved workshops and talks organized targeting various stakeholders in different settings and environments. Seven free workshops were conducted by the first author and the final author at the end of the research with targeted parent participants, educators, health practitioners, researchers, university students, and public audiences. Approximately 350 participants attended these workshops in total, which included approximately 40 participants who were parents of children with ASD. The venues were intentionally varied, including a school, university, community hall, and a large government hospital. These occurred in the Malaysian states of Kuala Lumpur, Selangor, and Negeri Sembilan. Feedback was gathered from the parent participants, who shared feeling that the themes well-represented their own experiences. At the university workshop, one of the study interviewees attended the event and described that having the opportunity to see the outcome of the study was very rewarding. In addition to the community engagement events, the innovative use of a different qualitative approach, IPA, for the pilot component and grounded theory for the current study allowed the researchers to have a more extensive understanding on finding meaning in the data and it helped heighten transparency in the analysis process (Frost et al., 2010).

### Procedure

#### Recruitment Strategy

Participants were recruited through various means; mainly through the researchers’ collaborative partnership with various ASD-related organizations, whereby contact was initiated via email and phone calls to center administrators. Hardcopy and online flyers for recruitment were posted to treatment centers, schools and online parent support groups. Potential participants were screened to assess if they met the inclusion and diagnostic criteria by one of the authors, who is a doctorally trained and licensed clinical psychologist (in both California and Malaysia) formally trained in ASD diagnostic assessment. The Social Responsive Scale-2 (SRS-2; Constantino and Gruber, 2012) was utilized to help confirm ASD diagnoses, with *T* scores equal or >60 used as an inclusion criterion for the ASD group. All participants in the current study had children who scored equal or >60 on the SRS-2. All diagnoses were performed by a registered professional (i.e., mental health professional or developmental pediatrician). The date of the diagnosis and name of the diagnosing professional was gathered for each child; the researchers confirmed the diagnosing professionals’ qualifications in the area. Prior to the interviews, explanatory statements describing the study and informed consent forms were read and signed by the participants. Parents also completed a history and demographic questionnaire.

#### Interview Process

Data were collected by the first author (during her Ph.D. study period) through face-to-face semi-structured interviews with open-ended questions. The first author had a Masters degree in Clinical Psychology and was a registered clinical psychologist with experience working with ASD. All interviews were audio-recorded with the participants’ consent. In addition, handwritten notations (field notes) were taken. The interviews with parent participants lasted 50–80 min (mean = 67.5 min). Most interviews were conducted at the participants’ houses. Four interviews were conducted at quiet restaurants, whereas two were conducted at the participants’ workplace and one at the researcher’s university. Interview location was chosen by the interviewees. Key events that were explored in the interviews included: early recognition of symptoms, the diagnosis process, treatment and interventions sought, experiences about parenting, the stressors and challenges faced, coping strategies, cultural influences, family and social support, as well as future recommendations to other parents, society and government (see the parent interview guide, Table 3).

**Table 3:** Interview guidelines for parents.

Parents interview guidelines
**Interview guide – interview topics, questions, and prompts**
(1) Please begin by telling me about your experience of having a child with ASD.
(a) Possible prompts regarding the diagnosis if not mentioned.
(i) Tell me about your experiences of first observing something of concern with your child.
(ii) Tell me about your experience of learning your child has an Autism Spectrum Disorder.
(iii) Tell me about the diagnosis process.
(2) Based on your experiences of raising a child with autism, what experiences have been most meaningful to you?
(3) Tell me about your experience of having a child with autism in your family.
(a) Tell me about how having a child with ASD has affected your family?
(b) What sort of behaviors affected your family the most?
(4) Tell me about the relationships between your child with Autism Spectrum Disorder and his/her siblings, you, your husband, and grandparents?
(a) How was your family affected?
(5) Tell me about any stressors or challenges, if any, that you have faced?
(a) Being a parent to a child with autism.
(b) Having a child with Autism Spectrum Disorder.
(c) Having a child with Autism Spectrum Disorder in your family.
(6) Tell me about what has helped you.
(a) Tell me about what has helped improve your well-being.
(b) Tell me about what has helped improve your family well-being.
(c) Tell me about what or who has helped or supported you.
(d) How have you and your family coped with having a child with Autism Spectrum Disorder?
(e) How much social support (e.g., from friends, relatives, support groups, neighbors, etc.) do you receive?
(f) How much has social support helped you cope?
(7) Tell me about the experience of being a parent to a child with Autism Spectrum Disorder in Malaysia and in your culture.
(8) Please describe the services, treatment, education that you have sought?
(9) What is your understanding of the development of your child’s condition?
(10) If you could provide advice or suggestions to other parents with a child with Autism Spectrum Disorder in Malaysia, what would it be? What advice or suggestions would you give to other parents to help improve their family well-being? What advice or suggestions would you like to give to mental health and health professionals in Malaysia?
(11) Please let me know any other comments that you have about this research topic. Please let me know any questions that you have about this research?


#### Data Analysis Process

All interviews were transcribed verbatim, read and compared with each other. On average, the researcher (first author) took 3–6 h to transcribe each interview. Most of the interviews were conducted in the Malay language given it is the first language that is spoken by most participants at home. This option reduced possible communication breakdowns and helped ensure that in-depth information was obtained. Phrases or statements (i.e., quotes) that were related to the current analysis were translated to English without changing its original meaning. Then, the translated quotes were checked for translation accuracy by another member of the research team, who like the first author also has excellent fluency in both English and Malay. Four interviews were conducted primarily in English, although occasional comments were made in Malay.

The interview transcripts were then imported to NVivo 10. The researchers had attended advanced training on qualitative software analysis tools. Following the grounded theory approach, the primary researcher read each transcript repeatedly before commencing the coding process. The initial analysis process began by labeling each response (open coding). Next, the initial codes were characterized into general categories (axial coding), aligned with the research question. To further analyze the data and identify themes, subcategories were developed within each broad category. The primary supervisor (last author) reviewed the codes and helped develop themes. A third-party researcher (see Marques and McCall, 2005) checked coding accuracy of one transcript. Then, the first author and co-authors discussed any disagreements of the suggested codes, before agreeing on the final codes and themes. All authors agreed on the final themes. All researchers were female. The fourth author’s field of specialization is Education (Special Education), and the other authors’ field of specialization is Psychology. The first and last author are trained and registered clinical psychologists. All authors hold doctoral degrees, with the first author completing her Ph.D. soon after completion of this present study.

#### Ethics Statement

This study was carried out in accordance with the recommendations of the Monash University Human Research Ethics Committee (MUHREC) and was conducted with permission from the participating organizations. All subjects gave written informed consent in accordance with the Declaration of Helsinki. The protocol was approved by MUHREC (CF12/1611-2012000868). Participation was voluntary and with written informed consent. Each parent was given a RM 50 (∼12 USD) shopping voucher. Names of all the participants and the names of any other individual referenced by the participants are pseudonyms to protect confidentiality. Income data is presented in aggregate form (not individually) to protect confidentiality: 40.9% of parent participants reported a personal income below RM3,000, 27.3% reported a personal income of RM3,000–RM5,000, and 31.8% reported a personal income above RM5,000.

According to the Department of Statistics Malaysia (2017a, 2017b), the median salary and wages of paid employees in 2016 was RM2,000 (RM2,000 for women and RM2,000 for men) and the median household income for Malaysians was RM5,228 in 2016. The currency conversion rate is approximately 1USD to 4RM. Personal income in this sample was similar to the median income in the country. However, this is likely insufficient to meet costs of care and afford private education for a child with ASD. Of concern, Kamaralzaman et al. (2018) identified in a sample of 245 Malaysian parents that the total cost of financing a child with ASD is approximately RM35,366 a year, a large and burdensome amount in Malaysia.

## Results

The study results were reported following the consolidated criteria for reporting qualitative research (COREQ) checklist (Tong et al., 2007; see Supplementary Table 1). Analyses of the participants’ interviews, yielded four prominent themes—“*initial reaction to child’s ASD symptoms and diagnosis,” “family life affected by a child with ASD,” “awareness about ASD in Malaysia,” and “coping strategies, wellbeing, and becoming resilient*”—that interact to foster growth and empowerment when adapting to a child with ASD in the family system. Table 4 illustrates the themes, subthemes and categories of the findings.

**Table 4:** Themes, subthemes, and categories.

Themes	Sub themes	Categories
Initial reaction to child’s ASD symptoms and diagnosis	Help seeking behavior	Proactive action Began the treatment prior to diagnosis. Delayed for further investigation and treatment
	Adjusting parents’ cognition, daily routines and life plans	Initial dismissal/Grief Redefine future dreams and hope Constant worries and anxious over future Positive self-thought and belief Busy daily living Redefine life priorities
	Stress due to the inadequate “system”	Limited awareness about autism Financial burden Difficulties in finding appropriate school, childcare, therapies and healthcare Shortage of qualified healthcare practitioners, teachers and caregivers Government policy and support on ASD Limited access due to logistic areas
Family life affected by a child with ASD	Parents’ emotional turmoil	Dealing with the symptomatic ASD condition Isolation due to child’s challenging behaviors Strain on family Job and career adjustments
	Impact on significant others	Siblings rivalry Burden on siblings and other family members
	Increase sense of connectedness	Family closeness Mutual relationship and understanding with others
Awareness of ASD in Malaysia	Lack of knowledge	Ignorance Inadequate understanding about ASD
	Judgemental environment	Bad parenting style and practices Stigma from others
	Pervasive cultural beliefs	Previous generations or parental sins Inadequate explanation and belief about the causes of ASD
Coping strategies, wellbeing and becoming resilient	From zero to hero	One little step at a time The power of knowledge Increase sense of competence
	Growing stronger together	Recognizing the joys Celebrating the little triumphs
	Stronger spiritual faith	Believe chosen by God Pure and innocent gift from God
	Social support	Immediate vs. extended family members Formal and informal support groups • Professionals, therapists and government • Community


### Theme 1: Initial Reaction to Child’s ASD Symptoms and Diagnosis

The first theme illustrates the parents’ initial reactions and understanding when they first learned about their child’s ASD symptoms and diagnosis.

#### Help Seeking Behaviors

The first subtheme explained how parents engaged in *help seeking behaviors* after they first noticed differences and/or delays in their child’s development. Most parents (54.5%) reported that they directly had taken proactive action, which included researching about autism on the internet, reading books and magazines, and seeking assessment from a specialist. Nasyran shared:

We wonder, is Nabila having a hearing problem? She just focused 100% to the television. My wife took her own initiative, she Googled a few websites to search for the information, and she got some information about autism symptoms…so it was her own initiative to find an answer.

Only a small number of parents (13.6%) went ahead and began treatment for their child prior to a formal diagnosis. Leeya spoke about her experience seeking alternative treatments and beginning interventions, while in queue on the long waiting list to see the pediatrician:

We brought her whenever anyone told us that this fellow can do this and this. I remember one fellow just pulled her hands and legs. I also don’t know what kind of treatment that is…(small laugh).

There were a number of parents (22.7%) who delayed further investigations and treatments due to reasons such as busy work schedules, cultural beliefs, “denial,” and obeying their families’ opinions. Hana recalled:

We noticed the difference when he was 3 years old, but most family members and friends kept telling, he still a young kid, so it is normal. Many of our friends and in fact relatives advised us to go and see a *‘bomoh’*^1^ first (small laugh). But we didn’t do that.

#### Adjusting Parents’ Cognition, Daily Routines, and Life Plans

Following diagnosis, the parents embarked on a “new journey” in parenting. Parents reported having to adjust many aspects of their life, including their cognitions, feelings, daily routines and their overall life plans. The majority of parents (72.7%) recalled that they initially went through the stages of grief and denial upon discovering their child’s diagnosis. Four out of eight fathers reported the feeling of “dismissal” and being in the denial stage for quite some time. Ramli shared:

My wife and I…we don’t expect to have a child with autism. We were shocked at the initial stage. Really shocked…(a bitter smile).

Nasyran expressed his sorrow:

Hmm (long pause and deep breath)…initially it’s tough…(pause) when the doctor said it is under OKU^2^ category. It is really tough for us…(long sigh).

The majority of parents (63.6%) also reported that since they learned about their child’s ASD diagnosis, their lives had been filled with anxiety over their child’s future. However, most parents (81.8%) reported that after a period of time, they readjusted their thoughts and beliefs about their child’s condition and future; and hence, became more positive and optimistic to persevere. The parents mentioned having to readjust their routines and life plans. Also, many parents (40.9%) expressed how they needed to redefine their priorities. Keen described, “Our family planning, first we thought we wanted three kids, but after we found out my eldest son’s case, so we stopped the idea and just concentrated on him.”

#### Stress Due to the Inadequate “System”

The third subtheme describes the stress that parents experience due to the inadequate “system.” “System” in this context was defined by parents as the existence (or lack thereof) of child-related agencies, either private or government-funded, that specifically accommodates the needs and development of a child with ASD. Inadequacy in the “system” included difficulties in finding appropriate schools, childcare therapies and healthcare; as well as a shortage of qualified healthcare practitioners, teachers, and caregivers for this group of children. Parents also described the financial burdens they face.

According to the majority of parents (63.6%), a contributing factor to this problem of stress is the limited awareness about autism in Malaysia. Several of the parents (31.8%) directly mentioned the poor governmental policy on ASD in Malaysia. Mawi expressed his opinion:

“All disabilities were mixed in the school. I think our government should look into this case more serious”.

Leeya (Mawi’s wife) added:

“Less places or centers that cater specifically for a child with autism. I think this is the government role.”

Many parents (45.5%) also described logistical problems for people who live in rural areas or in the outskirts of the city, where there are even more limited resources for interventions and more limited access to schools and therapy centers.

### Theme 2: Family Life Affected by a Child With ASD

The second theme illustrates how the sampled parents and their families have progressed with raising a child with ASD and describes how having a child with ASD in the family impacted the parents and their significant others.

#### Parents’ Emotional Turmoil

In the first subtheme, *parents’ emotional turmoil*, the majority of parents (81.8%) reported the difficulties they face in managing ASD symptomologies as one of the biggest challenges they face. Leena reiterated how her son’s rigidness triggered her stress:

He didn’t want to go to school, he sees the building, he starts screaming. Inside the car itself, he will start…he knows the road. His memory is very good *lah*. So, he knows we are taking the road, so he will already start crying. Inside the car, he will be pinching me, kicking me, I tell you…that jam itself is already stress, then behind me some other noise.

One father unhappily expressed how his sexual relationship is “disturbed” due to his daughter’s challenging behaviors, “She just want to sleep with us…she is very attached to me. It is a problem also…so my libido time is also NO. So how to spend my libido?” Another parent, Beetha, also spoke about the loss of spontaneity during family trips, due to Bevan’s difficult behavior.

Slightly more than half of the parents (59.1%) expressed feeling isolated as a consequence of their child’s challenging and difficult behaviors. Seven parents explained that they isolated themselves from family and friends because of their child’s behaviors. Leeya shared, “We decided not going to some social events, I don’t like the uneasy face that people made.” On the other hand, four parents described how other people excluded them and/or their child. Delina said, “I can see my nieces and nephews would exclude Rafiq from the group when we had a family gathering, as Rafiq is so disgusting to them…” (gloomy face, long sigh). Similarly, Camilah remembered how she felt isolated during a family gathering at her sister in-law’s house, “From their face reaction, I already can sense that they are not really welcome us. Maybe they just invited us as a matter of courtesy.”

Most of these experiences were mainly due to the child’s challenging behaviors, like “impulsivity.” Likewise, Camilah said, “There were many same incidents happened, you can see that people started to worry when you reached, as a little monster has come to destroy the event, to make chaos.”

Several parents (40.9%) also expressed how their child’s unexpected behavior puts a strain on their family life. Hana said, “Sometimes, when he’s back from school, he still didn’t know how to express his feelings. We were also tired, just came back from work, just out of blue, he throws tantrums, with the baby, another sister…quite stressful sometimes.”

Raising a child with ASD was also found to impact the parents’ employment and career. Some of the parents (45.5%) needed to readjust their working hours, shift from a full-time to part-time position, take unpaid leave, and/or even live separately from the family due to work commitments. Junaina explained, “I need to bring her to see OT, speech therapist, but I have very limited time. Every night before sleep, I asked myself, until when?”

Nasyran spoke about how his wife was negatively affected after he transferred to Peninsular Malaysia due to his work commitment, “Right now we are not living together, I am here alone and all of them are there, sometimes my wife did complain, she is the one who is taking care of all children plus Nabila, it is tough you know, but what to do? I need to grab this opportunity, then I can earn more to support them. Every night I would call her. When she talked to me at least relief her a bit.” Similarly, Keen lamented, “My husband is now working in East Malaysia, we are planning to live together there, but what I’m doing now is bringing my child back to KL for the therapies and schooling (sad face).”

#### Impact on Significant Others

Raising a child with ASD was also found to impact their significant others in many ways. Being a sibling to a child with ASD can be both a challenging and wisdom-developing experience. It was difficult for parents to allocate time to fulfill the needs of their other children. As such, parents occasionally experienced guilt. Suhaimi expressed, “Now my concern also on his eldest brother, because he always compares himself with Emran. Their age difference was only 1 year. After Emran was born, he got less attention. I can see that he is now, isolating himself from us and kept asking, why Emran always got more attention than me?” His wife, Delina also expressed, “Now he started complaining, everything Emran, why it is always Emran?”

Camilah talked about how Danish’s brother vented out his stress when their friends make fun of Danish at school, “He told me, I don’t like when my friends laughed at Danish. They make fun of Danish and called him ‘*budak cacat’* (child with mental disorder)”. Camilah mentioned she would continue to explain to Danish’s brother about Danish’s condition and encourage him to help his brother at school.

#### Increase Sense of Connectedness

On the other hand, the results showed that half of the parents (50%) perceived the positive impact that having a child with ASD brought to their relationship and understanding with their spouse and other family members. One clear finding is that the spouses felt the need to rely on one another more, and this brought them closer as a parent or couple to take care of their child with ASD. The family members would be more alert and aware of the needs and any little improvements showed by the child with ASD.

Nasyran - “When we brought her to my mother’s house, she has been treated like a ‘princess.’ Everyone is trying to take care of her. She made us closer and connect to each other.”

Ghaida - “I thank Allah, for me from the day he was born until today. He made our family relationship stronger, more bonded to each other.”

Mawi - “Having her made me have more time to communicate with my wife, yeah almost all the topics are related to her, but it increase our communication in a good way.”

### Theme 3: Awareness About ASD in Malaysia

The third theme illustrates how ASD is viewed within the social context in Malaysia. This theme also describes how differing ethnic groups, religious values, and family traditions may differently affect parental perceptions, stress, and coping strategies.

#### Lack of Knowledge

The first subtheme, lack of knowledge, is based on the mutual finding among all parents (100%) that while the awareness on autism is growing, there are still stigmas associated with ASD, and accordingly, there are still various misconceptions about ASD among the public in Malaysia. Two prominent categories emerged from this subtheme, which were ignorance and inadequate understanding about ASD.

A number of parents (38.8%), in fact, directly pointed out their own ignorance at the initial stages of the diagnosis. They expressed some remorse, by stating that they could have started the intervention earlier, and should have learned much more about their child’s condition, which could have helped them accept their child’s condition sooner. Reflecting back, Imran said, “I knew about autism quite late, when it happened to my son, then only we wanted to know more.” Farid similarly described, “Initially I was blank…I don’t know what is autism. I have no knowledge at all.” Likewise, Farena mentioned, “I think the awareness is still low, even myself I did not really know and bother about autism. When it happened to us now, then we started to bother. This is what happening in our society.”

Overall, all parents (100%) agreed that inadequate understanding about ASD in Malaysia is the most significant factor that contributed to the lack of awareness. It occurred within themselves, immediate and extended family members, the community, and the society as a whole. The word “autism” still remains a mystery to the general public as there seems to be little motivation for an individual to learn more about it unless they are affected by its conditions. Aneesa was explaining about her mom’s curiosity:

My mom kept asking, why she behaved like that, when would she be “normal” like her other grandchildren, is there any medication for her, when would she be cured… and the same questions keep going and going… until one point I just said to her, until she DIES mom… because I’m tired of explaining the same thing… (sighed with gloomy face).

Her husband, Ahmad, in turn explained how the community perceives autism:

Our community doesn’t understand…because she looks normal physically like other kids… we have to explain and explain most of the time. I said it is not related to a psychiatric condition and it is not a contagious disease.

Suhaimi shared his experience,

I brought him to Department of Education to discuss his schooling matter. We decided to put him in the special education class. The staff looked at us with puzzled expression and repeatedly said that he looked normal… I explained to them, he is an autistic boy. But I don’t think they really understand. That is what I can see in our society.

#### Judgmental Environment

The second subtheme explains how a judgmental environment contributes to the low awareness among the public in Malaysia. A number of parents (40.9%) described how they have been labeled as a parent who has bad parenting styles and practices. The manifestation of their child’s challenging behaviors, such as throwing tantrums and picky eating habits, are continuously being associated with bad parenting. Junaina stated, “Especially her picky eating… they questioned me why she must have her own and special food, and sort of accusation I felt sometimes when they said that I’m giving in too much to her, that’s why she became like that…”.

Razila shared her experience of when her son throws tantrums at the shopping complex:

Sometimes our community they always being good observers and commenters. They won’t help you much…yeah, there were some that would help, but most of the time, the kind of look that they gave you, especially when you have a child with special needs and they were throwing tantrums in public for instance… people would give you the unkindly stare and say, “Excuse me, do you know how to take care of kids?” (small laugh, long sigh).

Her husband, Ramli compared his experience of raising his child with ASD in Japan during his study leave in comparison to that in Malaysia. He described,

In Malaysia, we can see that people were not so comfortable with Rafiq’s tantrums and difficult behavior. From the way they stared at us, we know (smile). Even though we apologized and explained that he is OKU, people still complain. Compared to the Japanese, they also have little knowledge on autism, but the ways they approached us were different. They would try nicely to start a conversation and ask about the child, have you brought him for any treatment and so on and other nice questions.

Parents of children with ASD unfortunately, face social stigma from the public, which in turn makes their life more challenging. In this study, almost half of the parents (45.5%) reported how “social stigma” has affected their life. Mawi shared his experience of having to cope with such stigma from his superior at work:

It was the 1st day Laili is going to school, and I applied for leave earlier. She asked me, which of your child going to school? Is it the one who is disable (*cacat*)? I was really… argh (loud voice and sighed).

And he continued:

What really upset me, anything that related to my work, even my yearly performance appraisal, she would relate to my daughter’s condition…

Ramli shared his experience of managing Rafiq’s tantrum in the public:

I brought him for groceries, suddenly he started to cry and yelling. There was one Chinese uncle angrily stared at me and came to me and said, “Could you please bring your spoiled child to other place, I just want to enjoy my moments.”

#### Pervasive “Cultural Beliefs”

Another factor that influences the level of awareness among Malaysians is pervasive “cultural beliefs.” Some mothers directly described the beliefs of family members with regards to autism; whereby their child with ASD was seen as a consequence of previous wrongdoings of the parents and ancestors. They have been blamed for having “brought” autism into their families. Leena shared her experience:

That kind of comment sometimes just eat my mind why… why this is happening to me? What have I done until he became like this… your family doesn’t have any problem… you have no Down syndrome. When I first got to know that he has problem… they started to investigate my background. Are you having a problem? Or your family has a problem? All these really stress me out because they say that it could be your previous generation’s wrongdoing…

She continued sharing about her own father’s comment:

Indians they have these old beliefs and all that…Even my father…he used to comment to me his name is not nice, the day he was born is not a good day. All this until now they still pressure me. I still talk to my parents, but I just put them aside. I just tell them I already have accepted him. From the day one up till now, I’m the one taking care, nobody else, nobody else comes and visits me all that. So, it is fine to me. I don’t need…I’m not going to ask anybody’s help.

Similarly, Camilah shared her experience of people’s belief of her wrongdoings and sins, “Yes, there were people that said jokingly, maybe you have made many sins, so now you paid what you did” (sighed). On the other hand, Keen and her husband, who are free thinkers, nonetheless went to temples to seek explanations for their son’s condition. Keen further explained that, they did it not because they did not accept their child’s condition, but for their own peace of mind in knowing that she did the best that she could do. She said,

Yes, toward the things like that also, we will go and get some advice, because me and my husband…we didn’t have a specific belief or religion, but we went to temple and asked about this…what’s happening, what did we do wrong?

The last category on this subtheme describes how inadequate explanations and beliefs about the causes of ASD has in part contributed to pervasive cultural beliefs, and thus, the low awareness of ASD among Malaysians. Half of the parents (50%) in this study stated that the complexity of ASD symptoms makes it difficult for parents, family members, and the community to understand the causes of autism. This difficulty in turn, invites stigma, and leads to prejudiced behavior.

Aneesa spoke,

Many of my friends said my daughter has ‘the unseen friends’, that’s why she behaved differently… (laugh). And some people, especially a pregnant mother, they would try to avoid us because they are afraid it would infect their baby later, like the Malay say, ‘*Kenan’*^3^ or ‘*Badi’*^4^.

Ahmad, her husband added, “Our culture, for some people they believed this happened because you have “*saka*”^5^ even you tell it is not, but they would advise you, go and see “*bomoh*” (shaman).”

Ramli shared his experience with his own father and other extended family members:

Not all my relatives understand, even you have explained to them in a simple way about autism. Some of them still believe and relate to the mystic. And it is hard to change people’s belief.

Ramli then continued, “Even my own father, had a hard time to understand him. Even you have explained to him thousand times. He said to me, there is nothing in this world that has no cure. Go and seek alternative treatment…”. Nasyran shared his experience bringing his daughter for alternative treatment to see an “*ustaz*” (a pious religious man):

He explained to me about Amira’s condition is not because “*gangguan*” (being disturbed by the unseen spirit or ghost). She behaved like that because she has different aura. So, he gave me few verses from Quran for us to practice to soothe her emotion and behaviors.

### Theme 4: Coping Strategies, Wellbeing, and Becoming Resilient

The last theme illustrates how parents coped during the early process and continue to cope in this journey of raising a child with ASD.

#### From Zero to Hero

With the first subtheme, *from zero to hero*, parents reminisced of the initial stages, when they had just learned of the diagnosis. The majority of parents confessed that they had “zero” knowledge (i.e., no awareness) about “autism” until their own child exhibited symptoms of ASD and thereafter, was diagnosed. Nonetheless, as this subtheme illustrates the majority of parents *transformed themselves* to a “hero” despite struggling with the challenges of raising a child with ASD.

Three parents shared their thoughts on taking one step at a time. For example, Mawi said, “For us, we try to take things progressively. Right now, our focus is on her primary schooling. So, both of us only focus on the related activities and tasks for her primary school enrolments.” Likewise, Razila said, “You have to solve one by one, if not it would drive you crazy.” Lastly, Beetha shared her thoughts,

Just take it 1 day at a time. What drove me to seek all this knowledge is my desperation. I need something for Ryan. I need to sometimes educate (other) people and I need to know what he needs or wants and how to get it. If I can’t find people to get it, I have to get it (myself) in that situation. If I can’t find the people to deliver the services, I will need to equip myself to deliver the services, that’s my mentality I suppose.

More than half of the parents (68.2%) also explained about the power of knowledge in helping them cope. When parents were equipped with knowledge, it enabled them to develop a sense of competence. This could have subsequently enhanced their wellbeing and increased their resilience to face adversity. They also reported that knowing the facts and information of their child’s condition not only helped them in dealing with their child, but also acted as a foundation from which parents could inform and educate other family members, community, and the government about ASD.

Ghaida stated,

Nowadays, we have many platforms like ‘Autisme Malaysia’ on Facebook, and many more. They maybe can come out with a good approach to tell the parents, share with parents, and help each other in terms of education for the ASD children. We as a parent to this special child, we need explanation sometimes. Also, as a parent, we have to be more open-minded too about our child. And gain knowledge, the scope of knowledge is wide not only knowledge related to our child with ASD, but other things as well like how to handle other children, family, etc. It must be like a package… inclusive of all. And regarding our child’s education, don’t leave it to the teachers alone, sometimes we have to inform the teachers, share with them too, and work with them.

Six parents described how they empowered themselves by enrolling into a course related to special education, and simultaneously attended workshops and seminars. Ramli stated, “As a parent with a child with ASD, we must first be equipped with knowledge, go out and seek knowledge.” Similarly, two mothers, Beetha and Farena enrolled themselves into a course related to special education. Beetha mentioned:

I took up the advanced training in special education, took a whole course for 1.5 years because in between I had my fourth baby, so I did the whole course and I wanted to be better informed, and not be in doubt.

#### Growing Stronger Together

The second subtheme denotes how parents aim to grow stronger day after day. Half of the parents (50%) in this study had surprisingly viewed their child’s behavior problem as delightful and as a joy for them. Ghaida expressed, “His tantrum is a kind of therapy for us… I know it sounds a bit awkward. But that is what we feel…” (smile). Further, Delina expressed her thought about her son: “There is a moment where you ‘missed’ his part of tantrums when he was small. Because at that time everyone was giving attention to him” (small laugh).

Cherishing even a small achievement shown by a child with ASD helped to uphold parents’ sense of hope. The majority of parents (77.3%) reported that this practice helped them sustain and endure the challenges they experienced together, as Beetha expressed:

When your child actually makes eye contact with you and wants your attention for something, for his case, that is a major breakthrough, because otherwise he is not interested, he wants to do it just himself, but if he wants you to participate he actually wants you to join him or help him out, that’s the meaningful thing.

Razila similarly expressed how his son made his father feel content just through an instance of meaningful eye contact:

We had dinner together with the whole family members at my parents’ house. My father was asking my son, “Which type of food that you want” … and my son did not answer for sure but looked at him delightfully. I realized my father’s happiness face.

#### Stronger Spiritual Faith

The third subtheme explained how having and raising a child with ASD strengthened their spiritual faith, which consequently helped them get through the challenges faced. Half of the parents (50%) stated that God has given them a child with ASD not because of fate or any past sins or wrongdoings, rather they were specially chosen by God to take care and raise this special child. In addition, several parents (27.3%) reported a belief that a child with ASD is a pure and innocent gift from God.

Leena reflected, “Sometimes, you just sit down and think, what else can you do. We started going to church. The motivation came from God actually. Basically, God… God really push me to one real level actually.” Leeya stated, “This is our assignment…and we are selected mothers to have this kind of child.” Razila described her feeling,

I think Allah want to teach me something different by sending Rafiq to us. Sometimes, we make rules and laws, but we should bear in mind that they are only human laws, you must have toleration, understanding and loves. Those are things that I lack in myself before.”

Farena shared, “We sit down and think about him. Why our child? And my husband whispered to me, ‘Allah won’t bear us with something out of our capability’. From there, I need to move on and stay strong until now” (smile).

Suhaimi described how he felt blessed that his son was sent to him as an opportunity to gain more rewards in his afterlife:

I am a person that can accept everything, so like having him is an opportunity for all of us to go to *Jannah*^6^, opportunity to gain more “*pahala*” (reward from Allah), going to *Jannah* together. So, if I do not treat him nicely, I feel bad.

Ramli similarly shared his thoughts by saying:

“He is our ‘*saham’* (treasures). We already have a valuable treasure. So, if we take care of him and treat him nicely, our treasures will grow. Send him to learn Quran, it will be more growing.”

Finally, Nasyran said contentedly, “Autism is a gift, a valuable gift to parents, it is not a disaster.”

#### Social Support

The final subtheme, *social support*, explained how parents found that it is imperative to have a good support system from different parties. This not only helped the child with ASD to cope with his/her difficulties, but also helped parents improve their wellbeing and build resilience. The majority of parents (59.1%) described that they require support from immediate as well as extended family members. They reported that it takes a team to raise a child with ASD and that every member of the immediate family should be part of the child’s growth and development. Participation from extended family members was also found to be a support.

Half of the parents (50%) highlighted the importance of good communication between immediate family members and mutual understanding with their spouses. Farid said, “I would try my best to utilize the time that I have with my wife, we would discuss, do activities together, we need to create the situation, more understanding of each other, then we can help him.” His wife Ghaida added,

Also support from your spouse. Really important and helpful. Support that you only can get if you understand each other… you understand him and he understands you… you must have a mutual understanding on how to deal with the children. And I am lucky because my husband supports me more than 100%. Support doesn’t really mean you give a lot of money to your partner, but every second you try to understand and help each other, good communication, being with you in thick and thin.

They additionally reported that assistance from extended family members (e.g., grandmothers, in-laws and relatives) provided relief from being overwhelmed with numerous responsibilities. Beetha said, “If it weren’t (for) my mum’s help, I don’t think we could have done so much, she was very helpful, even though she cannot understand, she still wants to help a lot.” Keen similarly stated, “I got brothers stay nearby with me, my son very close with them.”

Most of the parents (77.3%) reported that formal and informal support groups were good sources of social support and gave them an opportunity to network with other parents helping them cope. Formal support groups include governmental and non-governmental organizations, as well as people who have specialized training. Parents also suggested “two-way communication” between them and health professionals or ASD related organizations to be very important. Particularly, it is important for health professionals and the staff of the organizations to include the parents as part of the team in caring for the child. Tahera said, “I’m so lucky, the school principal was very understanding. She always advises me to look at my son’s strengths rather his weaknesses. Most of the time she would share many tips, experience how to handle kids with autism.” Hana likewise said, “Like in NASOM (National Autism Society of Malaysia), all therapists are helpful, they would discuss with me many things and techniques about how to handle Hamdi.”

Parents also reported that they appreciate the government’s effort in providing some assistance for the OKU (people with disabilities). Nearly all the parents in this study registered their child with the Department of Social Welfare (JKM) under the category of person with disabilities. The child, therefore, has an identification card that is categorized under learning disabilities, which would also be applicable to children with Down syndrome, dyslexia, ADHD, etc.

Ahmad shared:

That OKU card is helpful sometimes, when she throws tantrums and we can’t control her, people started looking at you. Some people stare and some make faces… The security guard came to me and asked me to bring her out. I said to him, “My daughter got problem.” He still insisted me to remove Auni. And, I showed the OKU card to him. Then, he just walked away.

Camilah shared the same experience, “We just photocopy the card and at the back we write some information about autism.”

Parents further stated that they perceive the government to now be taking greater initiatives in helping children with autism as well as their families. Ramli said, “I heard there would be a specialized center that will be opened soon by the government for children with autism. I am really looking forward for that center.” Ghaida shared her experience of enrolling her son at the Community Rehabilitation Center,

Even though it is mixed with the other disabilities, there were a few activities that still benefited Ghazali. But I think the government should increase the salary of the teachers at the center. They are doing a great job but got less pay.

Suhaimi similarly shared her thoughts:

I can say the government is now more helpful, it is such a good movement. Now I can see the system in the special education class at the government school is improving. The teachers would provide one to one report in detail about all the students.

On the other hand, parents reported that informal support groups could come from anywhere in the community, such as other parents of a child with ASD, neighbors, religious groups, social welfare groups, etc. Sharing information and experiences with other parents, who have a child with ASD yielded many positive outcomes for the parents. For example, Hana elucidated, “You feel relief when you talk to the other parents that have a child with ASD too. In other words, share with people that in the same boat with you.”

Leena shared her experience on how the support group at her son’s school improved her wellbeing:

We met around 13 parents. We had a small group of parents and teachers where every 2 weeks we met the teachers. We went to the upstairs of the school and did activities together… So, there were a lot of stories. A lot of motivation from the teachers. We ate together, chit-chatted, exchanged small gifts. Oh, these 2 h really a good break for us (pleasant smile).

The majority of parents in this study, reported not only gaining psychological strength from social groups, rather they also used it as an opportunity to uptake the role of an advocate within the community. For instance, Ghaida expressed:

Being involved in the teacher–parent committee in my son’s school was such a golden opportunity. I have a space to educate the teachers and other parents about autism. It gave me hope and peace for my son.

There were several parents (22.7%) who directly expressed that “good people” still existed in the community. They described these members of the community as people who tried to understand and accept their child and his/her family unconditionally. Ahmad said, “The next door neighbor gave free WIFI to her. So just buy the laptop.” Likewise, Leena contentedly said, “We have nice people around here. … you don’t have it in the family but people around like friends, neighbors and all that, they are very nice to us” (smile).

## Discussion

This is one of the few studies that has, to date, explored the experiences, risk and protective processes of resilience in parents of children with ASD in Malaysia. Analyses of parent interviews in the present study revealed the dynamic mechanisms of becoming resilient in the Malaysian context. The first three themes–“*initial reaction to child’s ASD symptoms and diagnosis,” “family life affected by a child with ASD,”* and *“awareness about ASD in Malaysia”*–revolved around the (i) *stress and adversity* and the (ii) *adaptation and acceptance* of the parents. These two propositions describe the risk experience process of the parents. The last theme–*“coping strategies, wellbeing, and becoming resilient*”–highlights the (iii) *strengths and determination* of the parents and describes the protective experience process that helps them to build and maintain resilience.

### Stress and Adversity

The earlier themes found in this study illustrate the mechanism and dynamic process of resilience of the parents of children with ASD in the Malaysia context. Resilience refers to the person’s capacity to rebound and overcome trauma, negative experience and hardships (Garcia-Dia et al., 2013). Hence, from this perspective, one cannot be perceived as resilient unless he/she has faced some hardship and adversity. The findings support the conceptualization of resilience as a dynamic construct, similar to recent literature (Stainton et al., 2018).

In regard to the parent participants in this study, our findings describe how the diagnostic experiences of the majority of parents were marked by a series of difficulties, which were further exacerbated by limited resources in Malaysia. To a certain extent, the findings about the initial parents’ reactions and process toward child diagnosis were similar to previous studies in other countries (e.g., Myers et al., 2009; Giallo et al., 2013; Hodgson et al., 2016). However, the intense stress specific to the inadequate “system” such as healthcare, education and government systems and the limited support for ASD in this country could be due to the economic differences and limited involvement of the government. These factors were found to underlie the differences in how these processes were uniquely manifested and/or experienced in Malaysia. For instance, poorer policy and insufficient economic support for mental health and special needs in the region may play a role (e.g., Foronda, 2000; Ting and Chuah, 2010; Tait and Mundia, 2012; Foo et al., 2014; Ha et al., 2014; Ilias et al., 2017; Kamaralzaman et al., 2018). This situational factor may have intensified the difficulties and challenges faced by the parents in this country in comparison to those in a Western context.

Previous research supported the notion that differences of cultural beliefs, family traditions, and religious values contribute to the widespread prejudices and stigmas toward the child and family of children with ASD (Ghosh and Magana, 2009; Kang-Yi et al., 2013; Ha et al., 2014). The findings show that the majority of participants described their difficult experiences living in the judgmental environment and being labeled as a bad parent. A number of parents described how cultural beliefs influence the people’s understanding toward their child. The findings explain about how certain concepts or beliefs that are held by particular groups may negatively affect the perceptions of the people. The stigmas and false evaluation of the people, community and society at large can have a negative impact on the parents and family members. The findings highlight evidence that the negative emotional and social consequences due to stigma tend to increase the experiences of parenting stress.

Another prominent finding in Theme 3 was that the insufficient explanations and lay beliefs about ASD contribute to the low awareness about ASD in Malaysian society. Many Malaysian parents believe that having a child with ASD is related to “mystic” concepts. The beliefs that the child has the “unseen friend,” “*saka*,” or “*badi*” were common, especially among the Malay ethnic groups. Some of the people and family members have advised the parents to go and seek help from a “*bomoh*” or “*pawang*.” In another occasion, there was a group of people, especially pregnant mothers, that would avoid making contact with the child because they believed that it would “infect” their baby. These findings helped explain the culturally transmitted fears and concerns that they may have done something wrong in the past to cause the disorder (e.g., karma or spirit possession). This belief, which is relatively more prevalent in an Asian context, might influence how parents of children with ASD cope with challenges (e.g., Ting and Chuah, 2010; Rahman et al., 2012; Ha et al., 2014; Ilias et al., 2017).

### Adaptation and Acceptance

Adaptation to the child’s diagnosis and condition was the beginning of the healing process of parents and family members’ grief. The beginning of the journey was overwhelming and brought heartache to most of the parents. However, overtime, they learned and realized that the way they perceived the child’s condition and situation would powerfully influence the outcome or consequences. Accordingly, the majority of parents started to alter their *cognitions* and adjusted their routines and life plans. These processes were described by parents as acceptance and empowered them to be more vigilant in raising their child.

The findings in this current study also illustrate two different scenarios that have played out in family relationships over time. Initially, the majority of parents cited that dealing with the behavioral difficulties and ASD symptoms of their child with ASD was the most challenging parenting experience. The consequences of these difficulties would impact other family members as well, by creating misunderstanding, tension, and dissatisfaction, and reducing time spent with the other children (e.g., Angell et al., 2012; Nealy et al., 2012).

Furthermore, more than half of the parents reported the feeling of isolation that resulted from the challenging behaviors and ASD symptomologies that made them withdraw to avoid the unwanted trouble. This result was in turn related to stigma. Previous studies also found that the majority of parents that have a child with ASD may feel blamed for their child’s behaviors (Neely-Barnes et al., 2011), isolated and excluded from family and friends (Gray, 1993; Woodgate et al., 2008; Farrugia, 2009), and have an overall feeling of distress and burden because of stigma (Green, 2003).

On the other hand, nearly half of the parents directly perceived the positive impact that having a child with ASD brought to their relationship and understanding with their spouse and other family members. One clear finding is that the spouses felt that the need to rely on one another more brought them closer as a parent or couple. They also described that the presence of a child with ASD increased the sense of family *connectedness*. This result is similar to Rivers and Stoneman’s (2003) discussion of the importance of considering the family context and family wellbeing as contributing to the quality of sibling relationships in families with a child with ASD. At times, the child with ASD would be the center of attention for everyone in the family. The family members would be alert and aware of the needs and/or any little improvements showed by the child. This result supports the previous findings that families of children with ASD do not differ significantly from those without an ASD child in terms of cohesion and feelings of closeness (Altiere and von Kluge, 2009).

In addition to that, the identification of Walsh’s three domains of family resilience (the *family’s belief system*, *organizational skills*, and *communication processes*) were clearly evident in the participants’ interviews. For instance, a number of parents in this study expressed their positive experience raising a child with ASD and how this led to improvement in communication with their spouse and other family members, which acted as a buffer or protective factor against stress (Bayat, 2007; Ekas and Whitman, 2011). The findings also illustrated that having a child with ASD enhanced family cohesion within the nuclear as well as the extended family. The ability of the parents and other family members to make meaning out of the life changes that accompany raising a child with ASD is vital to the resilience development (Becvar, 2012). When parents and other family members accept the diagnosis and conditions of a child with ASD, the adaptation would further increase. The adaptation and acceptance is further related to the vital factors of strength and determination.

### Strengths and Determination

The abilities of parent participants to call forth and exhibit inner strength to proactively meet the personal challenges and manage adversities in relation to their child with ASD were important characteristics of resilience development. The study findings identified that despite the challenges faced, our parent participants have prevailed to accept the child unconditionally, cherishing the little joys and achievements and redefining their hopes and dreams which help them to developed strengths and determination.

The current findings also support previous studies that raising their child with ASD changed their life in a more positive way indirectly (Kayfitz et al., 2010; Bayat and Schuntermann, 2013). At times, they needed to redefine their dreams, hopes and expectations toward the child, but also many participants mentioned developing increased tolerance, patience, sensitivity toward others and self-advocacy skills in their everyday life. These results showed that the majority of parents in this study found positive meaning by raising a child with ASD. Findings supported previous studies that making meaning is a central aspect of coping with the adversity and resilient families build up from the ones who make meaning out of the adversity (Bayat, 2007; Myers et al., 2009).

The findings also revealed the good adaptive coping strategies of this group, such as thought reframing. It appeared that good coping strategies as suggested by the parents boosted their sense of purpose and in turn helped to build resilience. The findings further support previous studies’ recommendations that strengths and coping strategies are important indicators of a parent’s psychosocial well-being and result in reduced anger, anxiety and depression among parents of children with autism (Siah and Tan, 2016).

Along the journey, they described spiritual growth. The findings illustrate how parents learn and draw upon their faith following the diagnosis to help them make sense and construct meanings around the disorder. Their religious and spiritual beliefs played an important role in helping them to positively interpret and perceive the child’s disability. The belief that their child with ASD was a pure and innocent gift and they felt blessed that God chose them as a special parent was evident across the religions (i.e., Islam and Christianity). Supporting the current findings, previous research explained about how spiritual and religious beliefs influenced parents’ understanding and helped them in making positive interpretations about having a child with ASD (Tarakeshwar and Pargament, 2001; Ekas et al., 2009; Jegatheesan et al., 2010).

A few parents described that there was a group of “special people” in the community that were very supportive and understanding. In their explanation, these people are special because they accept the special child and the family members with an open heart and mind. A couple of parents described community acceptance as simply ‘understanding without being judgmental’ and ‘looking at the child with ASD as not being different but being the same as other children with different needs and different ways of thinking.’ Their acceptance and positive attitude brought happiness to the parents, improved their emotional wellbeing, and helped to build resilience. The findings can be related to the norms and courtesy of culture that anchor the Malaysian way of life. Although Malaysians may have different religious beliefs and practices, paying respect to and caring for people’s emotion, and being polite despite the differences, are part of the good virtue and vital concern for those people that live in the Malaysian multicultural country (Ramli, 2013).

Finally, the analyses further revealed the importance of society and governmental supports and resources (e.g., ASD parent support groups and online resources, school, therapy centers) to enhance parents’ resilience. The majority of parents agreed that currently there were some supports as well as resources provided by the government, however, there are still many gaps in terms of quality and accessibility. For instance, those parents that live in the sub-urban or rural areas would find difficulties in getting access to the places that offer intervention and face added struggles due to lack of resources. Those living in rural and even sub-urban areas face added risks and have less resources available to boost their resilience. The findings indicate that society and governmental support also plays a key role in building resilience, which therefore, necessitates the urgent need to increase support services to address the unmet needs of these parents on a nationwide level.

### Initial Theoretical Model of Resilience

**FIGURE 1:**
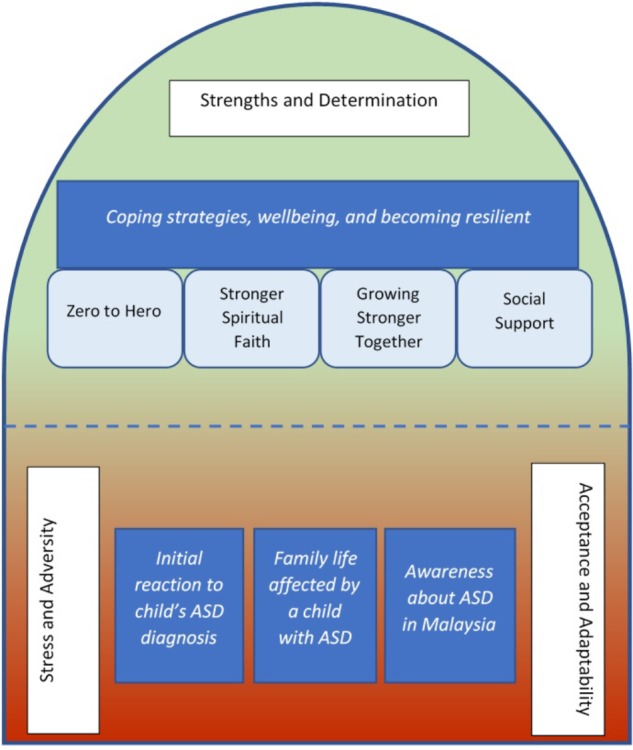
The dynamic mechanisms of resilience in parents of children in the Malaysian context.

Figure 1 illustrates the dynamic mechanisms of the initial theoretical model of resilience development. It illustrates the transformative experiences of parents of children with ASD in Malaysia, which could be translated as the risk and protective experiences throughout the process of resilience development. The figure depicts the road to resilience that involves the stress and adversities faced. The sources of stress come from different levels, such as from individuals, family members, the community, and society. Having a child with ASD may give tremendous and deep impacts due to the lifelong nature of the condition. Over time, the parents learned to accept and adapt with the situation. Accepting circumstances that cannot be changed enables parents to alter their cognitions and *adapt with* the stressful situation. These notions–“stress and adversity,” and “acceptance and adaptability”–are best described as the risk experiences of the parents of children with ASD that interplay in the process of resilience development.

Resilience is described as an ongoing process which requires time, constant effort and strong willpower from the individual and family. “Strengths and determination” is another notion that emerged from the findings and adds to this theoretical resilience model development. This notion is described as the protective experiences of the parents. With respect to the different coping strategies, these enable parents to deal with the stress and have a greater sense of control of their life.

There is a combination of factors at the different levels associated with resilience development. At the individual level, it involves thought, behavior, and action that can be learned, supporting the theoretical conception that resilience is malleable. The values and beliefs that are held by the individual parent also give impact to the process of resilience development. For instance, if a parent strongly believes that he/she was specially chosen by God, this belief lifts their sense of wellbeing and enhances resilience. Furthermore, the understanding, support and involvement of the family members and significant others enhance resilience development. Increased family cohesion helps build individual and family resilience. The social supports drawn from the community and society also help to promote resilience development. The risk and protective experiences are viewed as a dynamic, synergetic mechanisms rather than linear.

At this point, it is important to note that culture can influence and have impact on the resilience development process. How parents communicate and deal with the stress and adversities might be influenced by cultural differences. Family members and community understandings about the etiology of ASD and possible associated stigmas can have consequences and impact the parents. How other family members and the community connect themselves with the parents of children with ASD also might vary in respect to cultural practices and belief. Both *community risk* and *community resilience* processes are present. The findings in this current study theorized that the resilience of parents of children with ASD in the Malaysian context is best understood as the dynamic interplay between the risk and protective experiences of the parents. The process of resilience requires the functioning of many interacting systems within and around the parents and operating at different levels. These risk and protective experiences *blend* *together* (see Figure 1) resulting in transformative growth.

### Clinical Implications

The overall aims for the study were to address the lack of research to date exploring the experiences, risks and protective processes that contribute to parental stress and resilience for parents of children with ASD in the Malaysian setting. The application of the in-depth, constructive grounded theory qualitative approach contributes to the novel data obtained from parents. With reference to the Malaysian population, findings can help construct a more comprehensive theoretical understanding integrating a more expert understanding of the contextual environment.

The present study expands the knowledge base regarding the experiences, adaptation, wellbeing and resilience development of mothers and fathers of children with ASD in the Malaysian context. The findings from this study can help to inform about parents’ experiences in similar contexts (e.g., non-Western, Asian, collectivistic and developing countries) in terms of the differences in cultural, religious and spiritual beliefs and practices that might influence other people’s understanding and awareness. The findings lend support to the importance of acknowledging the culture-specific components that might influence how parents perceive, give meaning and adapt. It also shows how parents utilize certain coping strategies that might help them to grow through adversity. Therefore, the findings can be used to shape and guide the future clinical assessment and interventions to be used by healthcare and education professionals that tailor policy and services to the needs of the parents and the contextual environment.

The study’s inclusion of fathers helps expand the existing literature as some different emphasis areas were focused upon in the father interviews. The analyses of the transcripts indicated that most of the father participants expressed significant concern about the challenge of facing the behavioral issues exhibited by their child with ASD. Also, the findings described how fathers dealt with the difficult situation and learned to be self-aware and to control their emotions. Imran expressed,

I almost lost my control when he throws his tantrums after came back from school… just out of sudden… at that I time I also tired, just got back from office. If I just follow my irrational gut at that time… finish (small laugh).

Inclusion of both fathers and mothers in specialized parenting programs may help them to better manage a range of behavior problems common in children with ASD and help them increase their parenting skill self-efficacy. Also, fathers highlighted in somewhat greater depth their feelings of “denial” and “distancing” in the early period of noticing symptoms in their children. This observation points to the importance of actively involving both parents in the diagnostic and treatment planning process.

The study’s findings also help illustrate the truths about raising a child with ASD within the developing country where the “system” (e.g., health and education) for the children with ASD is still largely inadequate to provide good services. Greater attention needs to be placed upon the parents’ struggle to find and make use of the limited resources and supports available in Malaysia, especially in sub-urban and rural regions. Likewise, the shortage of qualified health practitioners, doctors, and teachers that have specified knowledge about ASD make the diagnostic process, therapies and treatment interventions tougher on the parents.

The findings provide strong implications for the Malaysian government to broaden autism awareness and services through different initiatives. For instance, in the health domain, extensive training should target all medical and allied health professionals, rather than just those who are specializing in ASD and related neuro-developmental disorders. In the education domain, more well-trained teachers are needed in teaching and educating children with ASD, and in helping to educate all children to learn more inclusive and accepting attitudes toward those with ASD, thereby helping build community resilience and reduce hurtful judgments and stigmas. Professionals across disciplines can be encouraged to increase the involvement of all family members during the assessment and intervention processes. Efforts to build resilience in the individual parents, the family system, and the community are recommended as applied initiatives in practice.

In a nutshell, this study has much potential significance to impact the local society and government. The findings make contributions in line with the Malaysian national priorities and objectives. A newfound commitment has arisen in top government circles, illustrating the current timely implications of the study. The findings in this study can serve as guidance for the Malaysian government in moving forward to plan services and education for those with ASD and their families.

The current study was unique, where the researchers took the initiative to conduct several workshops as an innovative and effective way of communicating and receiving feedback on the research findings. The underpinning rationale for these workshops assumed that the wider the audiences, the more opportunities to reach the community and create more awareness about ASD in the Malaysia.

### Limitations and Future Research

There are some limitations that are recommended to be addressed for future research. It is clear the parent participants in this study were Malaysian and not representative of parents in other cultural contexts. Furthermore, techniques of the participants’ recruitment for this study were purposive and utilized convenience sampling. Thus, the sampled mothers and fathers who volunteered for this study were likely those parents who had higher insight and motivation, further constraining the generalizability of the findings. However, it is worthwhile to mention that Malaysia shares common cultural traits and attributes with other Asian countries like Singapore, Indonesia, Vietnam, etc. Therefore, the findings illustrated in this study may be pertinent and applicable to families that share similar demographic backgrounds in these cultures. It is recommended for the future research to more cautiously identify the parents who are really struggling and those who are flourishing and observe over time the resilience process through interviews longitudinally. Future research can also make more detailed observations of participants’ sociodemographic background, which might influence how they shape their experiences. The largest proportion of the sample identified as Malay, and future research is recommended to explore in more depth other groups and the impact of their unique cultural views.

Also, the current findings were focused on parents whose children are of primary school age and we are uncertain how much our findings are relevant to parents whose children are in different stages of the developmental process (e.g., toddlers, adolescents, and adults). It is imperative and highly recommended for the future research to highlight the life experiences, challenges and coping of the parents across the developmental process as the challenges facing them may be different at every stage of child development. Especially, in Malaysia, there is an urgent need to provide services for adults with ASD and their families.

Additionally. our findings highlighted the importance of a family systems perspective to understanding resilience in mothers and fathers of children with ASD in Malaysia. Future research is recommended to explore in greater depth other family sub-systems. For example, the experience of the parents with respect to their other children and relationships between siblings are recommended focus areas. Additionally, inquiry exploring the quality of the relationship with professional caregivers and educators is important to develop a better understanding of the inter-disciplinary solutions to promote resilience development. The importance of adopting a holistic family centered approach is highlighted and the needs of all family members should be addressed and supported.

## Author Contributions

All authors listed have made a substantial, direct and intellectual contribution to the work, and approved it for publication.

## Conflict of Interest Statement

The authors declare that the research was conducted in the absence of any commercial or financial relationships that could be construed as a potential conflict of interest.

## Footnotes

1 Bomoh is a Malay shaman and traditional medicine practitioner.

2 OKU stands for *Orang Kurang Upaya*, which means individual with disability in the Malay language.

3 Kenan in this context refers to the presence of a peculiar or odd habit since birth.

4 Badi in this context refers to revenge in the form of illness from a spirit for harming or violating it.

5 Saka is the term that use to describe about spiritual possession in Malay.

6 Jannah in this context is refers to paradise according to Muslim belief.

## References

[B1] Allen K. A. Bowles T. V. Weber L. L. (2013). Mothers’ and fathers’ stress associated with parenting a child with autism spectrum disorder. *Autism Insights*, 5 1–11. 10.4137/AUI.S11094

[B2] Allen L. M. (2010). A critique of four grounded theory texts. *Qual. Rep.* 15 1606–1620. Available at: https://nsuworks.nova.edu/tqr/vol15/iss6/16

[B3] Altiere M. J. von Kluge S. (2009). Searching for acceptance: challenges encountered while raising a child with autism. *J. Intellect. Dev. Disabil.* 34 142–152. 10.1080/13668250902845202 19404835

[B4] American Psychiatric Association (2013). *Diagnostic and Statistical Manual of Mental Disorders*, 5th Edn Washington, DC: American Psychiatric Association 10.1176/appi.books.9780890425596

[B5] Angell M. E. Meadan H. Stoner J. B. (2012). Experiences of siblings of individuals with autism spectrum disorders. *Autism Res. Treat.* 2012:949586. 10.1155/2012/949586 22928104 PMC3420668

[B6] Bayat M. (2007). Evidence of resilience in families of children with autism. *J. Intellect. Disabil. Res.* 51 702–714. 10.1111/j.1365-2788.2007.00960.x 17845239

[B7] Bayat M. Schuntermann P. (2013). “Enhancing resilience in families of children with autism spectrum disorder”, in *Handbook of Family Resilience* ed. Becvar D. S. (New York, NY: Springer), 409–424. 10.1007/978-1-4614-3917-2_23

[B8] Becvar D. S. (2012). *Handbook of Family Resilience.* Berlin: Springer Science and Business Media.

[B9] Bekhet A. K. Johnson N. L. Zauszniewski J. A. (2012). Effects on resilience of caregivers of persons with autism spectrum disorder: the role of positive cognitions. *J. Am. Psychiatr. Nurses Assoc.* 18 337–344. 10.1177/1078390312467056 23139377

[B10] Bogner A. Littig B. Menz W. (eds). (2009). *Interviewing Experts (Research Methods Series).* Basingstoke: Palgrave Macmillan Limited 10.1057/9780230244276

[B11] Braunstein V. L. Peniston N. Perelman A. Cassano M. C. (2013). The inclusion of fathers in investigations of autistic spectrum disorders. *Res. Autism Spectr. Disord.* 7:7 10.1016/j.rasd.2013.03.005

[B12] Charmaz K. (2000). “Constructivist and objectivist grounded theory” in *Handbook of Qualitative Research*, eds Denzin N. Lincoln Y. (Thousand Oaks, CA: SAGE Publications Inc).

[B13] Charmaz K. (2006). *Constructing Grounded Theory: a Practical Guide through Qualitative Analysis.* London: Sage Publications Ltd.

[B14] Compas B. E. Malcarne V. L. Fondacaro K. M. (1988). Coping with stressful events in older children and young adolescents. *J. Consult. Clin. Psychol.* 56 405–411. 10.1037/0022-006X.56.3.405 3397433

[B15] Constantino J. N. Gruber C. P. (2012). *Social Responsiveness Scale, (SRS-2).* Torrance, CA: Western Psychological Services.

[B16] Creswell J. W. (1998). *Qualitative Inquiry and Research Design: Choosing Among Five Traditions.* Thousand Oaks, CA: Sage.

[B17] Creswell J. W. (2007). *Qualitative Inquiry and Research Method: Choosing Among Five Approaches*, 2nd Edn Thousand Oaks, CA: Sage.

[B18] Cridland E. K. Jones S. C. Magee C. A. Caputi P. (2014). Family-focused autism spectrum disorder research: a review of the utility of family systems approaches. *Autism* 18 213–222. 10.1177/1362361312472261 24092840

[B19] Daley T. C. Singhal N. Krishnamurthy V. (2013). Ethical considerations in conducting research on autism spectrum disorders in low and middle income countries. *J. Autism Dev. Disord.* 43 2002–2014. 10.1007/s10803-012-1750-2 23283629

[B20] Dyches T. T. Wilder L. K. Sudweeks R. R. Obiakor F. E. Algozzine B. (2004). Multicultural issues in autism. *J. Autism Dev. Disord.* 34 211–222. 10.1023/B:JADD.0000022611.80478.73 15162939

[B21] Ekas N. V. Whitman T. L. (2011). Adaptation to daily stress among mothers of children with an autism spectrum disorder: the role of daily positive affect. *J. Autism Dev. Disord.* 41 1202–1213. 10.1007/s10803-010-1142-4 21125322

[B22] Ekas N. V. Whitman T. L. Shivers C. (2009). Religiosity, spirituality, and socioemotional functioning in mothers of children with autism spectrum disorder. *J. Autism Dev. Disord.* 39 706–719. 10.1007/s10803-008-0673-4 19082877

[B23] Falk N. H. Norris K. Quinn M. G. (2014). The factors predicting stress, anxiety and depression in the parents of children with autism. *J. Autism Dev. Disord.* 44 3185–3203. 10.1007/s10803-014-2189-4 25022253

[B24] Farrugia D. (2009). Exploring stigma: medical knowledge and the stigmatisation of parents of children diagnosed with autism spectrum disorder. *Soc. Health Illn.* 31 1011–1027. 10.1111/j.1467-9566.2009.01174.x 19659737

[B25] Fenwick-Smith A. Dahlberg E. E. Thompson S. C. (2018). Systematic review of resilience-enhancing, universal, primary school-based mental health promotion programs. *BMC Psychol.* 6:30. 10.1186/s40359-018-0242-3 29976252 PMC6034212

[B26] Firth I. Dryer R. (2013). The predictors of distress in parents of children with autism spectrum disorder. *J. Intellect. Dev. Disabil.* 38 163–171. 10.3109/13668250.2013.773964 23509963

[B27] Fontil L. Petrakos H. H. (2015). Transition to school: the experiences of Canadian and immigrant families of children with autism spectrum disorders. *Psychol. Sch.* 52 773–788. 10.1002/pits.21859

[B28] Foo M. Yap P. M. E. H. Sung M. (2014). The experience of Singaporean caregivers with a child diagnosed with autism spectrum disorder and challenging behaviours. *Qual. Soc. Work* 14:5 10.1177/1473325014558662

[B29] Foronda C. G. (2000). Coping mechanism of women as solo parents of children with autism. *Rev. Womens Stud.* 10 69–95.

[B30] Freeth M. Milne E. Sheppard E. Ramachandran R. (2014). “Autism across cultures: perspectives from non-western cultures and implications for research,” in *Handbook of Autism and Pervasive Developmental Disorders*, ed. Volkmar F.R. Rogers S. Paul R. Pelphrey K. (Hoboken, NJ: Wiley), 997–1013.

[B31] Frost N. Nolas S. M. Brooks-Gordon B. Esin C. Holt A. Mehdizadeh L. (2010). Pluralism in qualitative research: the impact of different researchers and qualitative approaches on the analysis of qualitative data. *Qual. Res.* 10 441–460. 10.1186/s13012-014-0175-z 25417095 PMC4247765

[B32] Fusch P. I. Ness L. R. (2015). Are we there yet? Data saturation in qualitative research. *Qual. Rep.* 20 1408–1416.

[B33] Garcia-Dia M. J. DiNapoli J. M. Garcia-Ona L. Jakubowski R. O’flaherty D. (2013). Concept analysis: resilience. *Arch. Psychiatr. Nurs.* 26 264–270. 10.1016/j.apnu.2013.07.003 24238005

[B34] Ghosh S. Magana S. (2009). A rich mosaic: emerging research on Asian families of persons with intellectual and developmental disabilities. *Int. Rev. Res. Ment. Retard.* 37 179–212. 10.1016/S0074-7750(09)37006-8

[B35] Giallo R. Wood C. E. Jellett R. Porter R. (2013). Fatigue, wellbeing and parental self-efficacy in mothers of children with an autism spectrum disorder. *Autism* 17 465–480. 10.1177/1362361311416830 21788255

[B36] Gray D. E. (1993). Negotiating autism: relations between parents and treatment staff. *Soc. Sci. Med.* 36 1037–1046. 10.1016/0277-9536(93)90121-J 8475419

[B37] Gray D. E. (2002a). ‘Everybody just freezes. Everybody is just embarrassed’: felt and enacted stigma among parents of children with high functioning autism. *Soc. Health Illn.* 24 734–749. 10.1111/1467-9566.00316

[B38] Gray D. E. (2002b). Ten years on: a longitudinal study of families of children with autism. *J. Intellect. Dev. Disabil.* 27 215–222. 10.1007/s10803-011-1198-9 21347614

[B39] Green S. E. (2003). “What do you mean ‘what’s wrong with her?”: stigma and the lives of families of children with disabilities. *Soc. Sci. Med.* 57 1361–1374. 10.1016/S0277-9536(02)00511-7 12927467

[B40] Ha V. S. Whittaker A. Whittaker M. Rodger S. (2014). Living with autism spectrum disorder in Hanoi, Vietnam. *Soc. Sci. Med.* 120 278–285. 10.1016/j.socscimed.2014.09.038 25262315

[B41] Hawley D. R. DeHaan L. (1996). Toward a definition of family resilience: integrating life span and family perspectives. *Fam. Process* 35 283–298. 10.1111/j.1545-5300.1996.00283 9111710

[B42] Hayes S. A. Watson S. L. (2013). The impact of parenting stress: a meta-analysis of studies comparing the experience of parenting stress in parents of children with and without autism spectrum disorder. *J. Autism Dev. Disord.* 43 629–642. 10.1007/s10803-012-1604-y 22790429

[B43] Herring S. Gray K. Taffe J. Tonge B. Sweeney D. Einfeld S. (2006). Behaviour and emotional problems in toddlers with pervasive developmental disorders and developmental delay: associations with parental mental health and family functioning. *J. Intellect. Disabil. Res.* 50(Pt 12), 874–882. 10.1111/j.1365-2788.2006.00904.x 17100948

[B44] Hodgson A. R. Freeston M. H. Honey E. Rodgers J. (2016). Facing the unknown: intolerance of uncertainty in children with autism spectrum disorder. *J. Appl. Res. Intellect. Disabil.* 30 336–344. 10.1111/jar.12245 26868412

[B45] Ilias K. Liaw J. H. H. Cornish K. Park M. Golden K. J. (2017). Well-being of mothers of children with “A¬-U-¬T-¬I-¬S-¬M” in Malaysia: an interpretative phenomenological analysis study. *J. Intellect. Dev. Disabil.* 42 74–89. 10.3109/13668250.2016.1196657

[B46] Ilias K. Cornish K. Kummar A. S. Park M. S.-A. Golden K. J. (2018). Parenting stress and resilience in parents of children with autism spectrum disorder (ASD) in Southeast Asia: a systematic review. *Front. Psychol.* 9:280. 10.3389/fpsyg.2018.00280 29686632 PMC5900388

[B47] Jegatheesan B. Miller P. J. Fowler S. A. (2010). Autism from a religious perspective: a study of parental beliefs in South Asian Muslim immigrant families. *Focus Autism Other Dev. Disabil.* 25 98–109. 10.1177/1088357610361344

[B48] Jones L. Totsika V. Hastings R. P. Petalas M. A. (2013). Gender differences when parenting children with autism spectrum disorders: a multilevel modeling approach. *J. Intellect. Dev. Disabil.* 43 2090–2098. 10.1007/s10803-012-1756-9 23307420

[B49] Kamaralzaman S. Toran H. Mohamed S. Abdullah N. B. (2018). The economic burden of families with autism spectrum disorders (ASD) children in Malaysia. *J. ICSAR*, 2 71–77. Available at: http://journal2.um.ac.id/index.php/icsar/article/viewFile/2253/1608

[B50] Kang-Yi C. D. Grinker R. R. Mandell D. S. (2013). Korean culture and autism spectrum disorders. *J. Autism Dev. Disord.* 43 503–520 10.1007/s10803-012-1570-4 22723126

[B51] Kayfitz A. D. Gragg M. N. Robert Orr R. (2010). Positive experiences of mothers and fathers of children with autism. *J. Appl. Res. Intellect. Disabil.* 23 337–343. 10.1111/j.1468-3148.2009.00539.x

[B52] Keshavarz S. Baharudin R. (2009). Parenting style in a collectivist culture of Malaysia. *Eur. J. Soc. Sci.* 10 66–73. 23991523 10.1080/00221325.2012.678419

[B53] Kitayama S. Park H. Sevincer A. T. Karasawa M. Uskul A. K. (2009). A cultural task analysis of implicit independence: comparing North America, Western Europe, and East Asia. *J. Pers. Soc. Psychol.* 97 236–255. 10.1037/a0015999 19634973

[B54] Kuhn J. C. Carter A. S. (2006). Maternal self-efficacy and associated parenting cognitions among mothers of children with autism. *Am. J. Orthopsychiatry* 76 564–575. 10.1037/0002-9432.76.4.564 17209724

[B55] Lam A. G. Zane N. W. (2004). Ethnic differences in coping with interpersonal stressors a test of self-construals as cultural mediators. *J. Cross Cult. Psychol.* 35 446–459. 10.1177/0022022104266108

[B56] Lazarus R. S. Folkman S. (1984). *Stress Appraisal and Coping.* New York, NY: Springer.

[B57] Lecavalier L. Leone S. Wiltz J. (2006). The impact of behaviour problems on caregiver stress in young people with autism spectrum disorders. *J. Intellect. Disabil. Res.* 50(Pt 3), 172–183. 10.1111/j.1365-2788.2005.00732.x 16430729

[B58] Leone E. Dorstyn D. Ward L. (2016). Defining resilience in families living with neurodevelopmental disorder: a preliminary examination of Walsh’s framework. *J. Dev. Phys. Disabil.* 28 595–608. 10.1007/s10882-016-9497-x

[B59] Marques J. F. McCall C. (2005). The application of interrater reliability as a solidification instrument in a phenomenological study. *Qual. Rep.* 10 439–462.

[B60] Mash E. J. Johnston C. (1983). Parental perceptions of child behavior problems, parenting selfesteem, and mother’s reported stress in younger and task situations. *J. Clin. Child Psychol.* 12 337–346. 10.1037//0022-006x.51.1.86 6826870

[B61] McConnell D. Savage A. Breitkreuz R. (2014). Resilience in families raising children with disabilities and behavior problems. *Res. Dev. Disabil.* 35 833–848. 10.1016/j.ridd.2014.01.015 24491480

[B62] McStay R. L. Dissanayake C. Scheeren A. Koot H. M. Begeer S. (2014). Parenting stress and autism: the role of age, autism severity, quality of life and problem behaviour of children and adolescents with autism. *Autism* 18 502–510. 10.1177/1362361313485163 24104515

[B63] Meadan H. Halle J. W. Ebata A. T. (2010). Families with children who have autism spectrum disorders: stress and support. *Except. Child.* 77 7–36. 10.1177/001440291007700101

[B64] Meirsschaut M. Roeyers H. Warreyn P. (2010). Parenting in families with a child with autism spectrum disorder and a typically developing child: mothers’ experiences and cognitions. *Res. Autism Spectr. Disord.* 4 661–669. 10.1016/j.rasd.2010.01.002

[B65] Ministry of Health Malaysia (2014). *Ministry of Health Clinical Practice Guidelines: Management for Autism Spectrum Disorder in Children and Adolescents.* Available at: www.moh.gov.my

[B66] Morse J. M. Niehaus L. (2009). *Mixed Method Design: Principles and Procedures.* Walnut Creek, CA: Left Coast Press 10.1016/j.rasd.2009.01.004

[B67] Myers B. J. Mackintosh V. H. Goin-Kochel R. P. (2009). “My greatest joy and my greatest heart ache”: parents’ own words on how having a child in the autism spectrum has affected their lives and their families’ lives. *Res. Autism Spectr. Disord.* 3 670–684. 10.1080/10522158.2012.675624

[B68] Nealy C. E. O’Hare L. Powers J. D. Swick D. C. (2012). The impact of autism spectrum disorders on the family: a qualitative study of mothers’ perspectives. *J. Fam. Soc. Work* 15 187–201. 10.1080/10522158.2011.571539

[B69] Neely-Barnes S. L. Hall H. R. Roberts R. J. Graff J. C. (2011). Parenting a child with an autism spectrum disorder: public perceptions and parental conceptualizations. *J. Fam. Soc. Work* 14 208–225.

[B70] Neik T. T. X. Lee L. W. Low H. M. Chia N. K. H. Chua A. C. K. (2014). Prevalence, diagnosis, treatment and research on autism spectrum disorders (ASD) in Singapore and Malaysia. *Int. J. Spec. Educ.* 29 82–92. 10.2147/NDT.S100634 27103804 PMC4827600

[B71] Ooi K. L. Ong Y. S. Jacob S. A. Khan T. M. (2016). A meta-synthesis on parenting a child with autism. *Neuropsychiatr. Dis. Treat.* 12 745–762. 10.2147/NDT.S100634 27103804 PMC4827600

[B72] Patterson J. M. (2002). Integrating family resilience and family stress theory. *ıJ. Marriage Fam.* 64 349–360.

[B73] Quintero N. McIntyre L. L. (2010). Sibling adjustment and maternal well-being: an examination of families with and without a child with an autism spectrum disorder. *Focus Autism Other Dev. Disabl.* 25 37–46. 10.5348/ijcri-2012-06-138-CR-11 21037802 PMC2966315

[B74] Rahman F. N. A. Ismail W. S. W. Jaafar N. R. N. Fong L. S. Sharip S. Midin M. (2012). Reducing the isolation: a Malaysian family in need. *Int. J. Case Rep. Imag*es 3 46–49. 10.5348/ijcri-2012-06-138-CR-11

[B75] Ramli R. (2013). Culturally appropriate communication in Malaysia: *budi bahasa* as warranty component in Malaysian discourse. *J. Multicult. Discour.* 8 65–78. 10.1007/s10826-011-9477-9

[B76] Ravindran N. Myers B. J. (2012). Cultural influences on perceptions of health, illness, and disability: a review and focus on autism. *J. Child Fam. Stud.* 21 311–319. 10.5539/ass.v9n12p261

[B77] Razali N. M. Toran H. Kamaralzaman S. Salleh N. M. Yasin M. H. M. (2013). Teachers’ perceptions of including children with autism in a preschool. *Asian Soc. Sci.* 9 261–267. 10.1023/A:1025006727395

[B78] Rivers J. W. Stoneman Z. (2003). Sibling relationships when a child has autism: marital stress and support coping. *J. Autism Dev. Disord.* 33 383–394. 10.1155/2011/145359 12959417

[B79] Samadi S. A. McConkey R. (2011). Autism in developing countries: lessons from Iran. *Autism Res. Treat.* 2011:145359. 10.1155/2011/145359 22937242 PMC3420542

[B80] Selin H. (ed.). (2013). *Parenting. (Across )Cultures: Childrearing, Motherhood and Fatherhood in Non-Western Cultures*, Vol. 7. Berlin: Springer Science and Business Media 10.5463/dcid.v27i1.485

[B81] Siah P. C. Tan S. H. (2016). Relationships between sense of coherence, coping strategies and quality of life of parents of children with autism in Malaysia: a case study among Chinese parents. *Disabil. CBR Inclusive Dev.* 27 78–91. 10.1111/eip.12726 30126047

[B82] Stainton A. Chisholm K. Kaiser N. Rosen M. Upthegrove R. Ruhrmann S. (2018). Resilience as a multimodal dynamic process. *Early Interv. Psychiatry* 10.1111/eip.12726 [Epub ahead of print]. 30126047

[B83] Stoner J. B. Angell M. E. House J. J. Bock S. J. (2007). Transitions: perspectives from parents of young children with autism spectrum disorder (ASD). *J. Dev. Phys. Disabil.* 19 23–39.

[B84] Tait K. J. Mundia L. (2012). The impact of a child with autism on the Bruneian family system. *Int. J. Spec. Educ.* 27 199–212. 10.1177/108835760101600408

[B85] Tarakeshwar N. Pargament K. I. (2001). Religious coping in families of children with autism. *Focus Autism Other Dev. Disabl.* 16 247–260.

[B86] Ting S. H. Chuah H. K. (2010). Parents’ recognition of autistic behaviour and their coping strategies: a case study at Sarawak Autistic Association. *Malays. J. Soc. Policy Soc.* 7 52–65. 10.1016/S0891-4222(02)00099-9

[B87] Tobing L. E. Glenwick D. S. (2002). Relation of the childhood autism rating scale-parent version to diagnosis, stress, and age. *Res. Dev. Disabl.* 23 211–223. 12102589 10.1016/s0891-4222(02)00099-9

[B88] Tong A. Sainsbury P. Craig J. (2007). Consolidated criteria for reporting qualitative research (COREQ): a 32-item checklist for interviews and focus groups. *Int. J. Qual. Health Care* 19 349–357. 10.1093/intqhc/mzm042 17872937

[B89] Toran H. (2011). Experience and challenges in setting up a model demonstration classroom for children with autism in Malaysia. *Int. J. Educ. Admin. Dev.* 22 37–47.

[B90] Walsh F. (1998). *Strengthening Family Resilience.* New York, NY: Guilford Press.

[B91] Walsh F. (2003). Family resilience: a framework for clinical practice. *Fam. Process* 42 1–18 12698595 10.1111/j.1545-5300.2003.00001.x

[B92] Walsh F. (2016). *Strengthening Family Resilience*, 3rd Edn New York, NY: Guilford Press.

[B93] Weiss J. A. Cappadocia M. D. MacMullin J. A. Viecili M. Lunsky Y. (2012). The impact of child problem behaviours on children with ASD on parent mental health: the mediating role of acceptance and empowerment. *Autism* 16 261–274. 10.1177/1362361311422708 22297202

[B94] Woodgate R. L. Ateah C. Secco L. (2008). Living in a world of our own: the experience of parents who have a child with autism. *Qual. Health Res.* 18 1075–1083. 10.1177/1049732308320112 18650563

[B95] Xue J. Ooh J. Magiati I. (2014). Family functioning in Asian families raising children with autism spectrum disorders: the role of capabilities and positive meanings. *J. Intellect. Disabil. Res.* 58 406–420. 10.1111/jir.12034 23510076

