# Supplementary material for: Risk and Resilience Among Mothers and Fathers of Primary School Age Children With ASD in Malaysia: A Qualitative Constructive Grounded Theory Approach
Source: Front Psychol. 2019 Jan 8;9:2275. doi: 10.3389/fpsyg.2018.02275 (PMC6331527; doi:10.3389/fpsyg.2018.02275)
Supplement: Supplementary file 1 [file Table_1.pdf]

Supplementary Table 1

Manuscript:

Risk and Resilience among Mothers and Fathers of Primary School Age Children with ASD in Malaysia: A Qualitative Constructive Grounded Theory Approach

Kartini Ilias, Kim Cornish, Miriam S. Park, Hasnah Toran and Karen J. Golden

Consolidated criteria for reporting qualitative studies (COREQ): 32-item checklist

Developed from:
Tong, A., Sainsbury, P., and Craig, J. (2007). Consolidated criteria for reporting qualitative research (COREQ): a 32-item checklist for interviews and focus groups. *Int. J. Qual. Health Care 19*, 349–357. doi: 10.1093/intqhc/mzm042

No. Item	Guide questions/ description	Reported on Page #	Additional information
**Domain 1: Research team and reflexivity**			
Personal Characteristics			
1. Interviewer/ facilitator	Which author/s conducted the interview or focus group?	Page 6	Kartini Ilias conducted the 22 parent interviews. Karen J. Golden conducted the key informant interview described in this article. Kartini Ilias conducted 5 additional key informant interviews not described in this current article.
2. Credentials	What were the researcher's credentials? E.g. PhD, MD	Page 1, 6, and 7	
3. Occupation	What was their occupation at the time of the study?	Page 1 and 6	Kartini Ilias – PhD Student at Monash University Malaysia and Senior Lecturer at Universiti Teknologi MARA, Puncak Alam, Malaysia,<br><br>Karen J. Golden – Senior Lecturer at Monash University Malaysia

			Kim Cornish – Head, School of Psychological Sciences, Monash University and Executive Director at Monash Institute of Cognitive and Clinical Neurosciences (MICCN)<br><br>Miriam S. Park – Senior Lecturer at Monash University Malaysia<br><br>Hasnah Toran – Associate Professor at Faculty of Education, Universiti Kebangsaan Malaysia and Autism Centre Director at Permata Kurnia Centre
4. Gender	Was the researcher male or female?	Page 7	
5. Experience and training	What experience or training did the researcher have?	Page 6 and 7	
Relationship with participants			
6. Relationship established	Was a relationship established prior to study commencement?	Page 6 and 7	The first, fourth and fifth author had a collaborative partnership with some ADS-related organizations from past research projects. However, there was no relationship established between the interviewer and participants prior to the study commencement.
7. Participant knowledge of the interviewer	What did the participants know about the researcher? e.g. personal goals, reasons for doing the research	Page 6 and 7	Participants were aware that the research was for a doctorate study. Participants did get information from the researcher about the study, such as the goals and reasons for the research, in the detailed explanatory statement. Participants read the written explanatory statement and signed the written informed consent forms prior to the interview.
8. Interviewer characteristics	What characteristics were reported about the inter viewer/facilitator? e.g. Bias, assumptions, reasons and interests in the research topic	Page 6 and 7	

**Domain 2: study design**			
Theoretical framework			
9. Methodological orientation and Theory	What methodological orientation was stated to underpin the study? e.g. grounded theory, discourse analysis, ethnography, phenomenology, content analysis	Page 1, 3, 4, and 5	
Participant selection			
10. Sampling	How were participants selected? e.g. purposive, convenience, consecutive, snowball	Page 6 and 17	
11. Method of approach	How were participants approached? e.g. face-to-face, telephone, mail, email	Page 3, 4, and 6	
12. Sample size	How many participants were in the study?	Page 3, 4, and 5	22 parent interviews<br><br>1 professional key informant interview, 350 participants in community engagement events<br><br>An additional 5 professional key informants were interviewed but this was not a focus nor described in the present article.
13. Non-participation	How many people refused to participate or dropped out? Reasons?	Page 6	All participants who expressed interest to be interviewed participated in the study. All participants' children met the inclusion criteria.
Setting			
14. Setting of data collection	Where was the data collected? e.g. home, clinic, workplace	Page 6	Setting and language used were both described on page 6.

15. Presence of non-participants	Was anyone else present besides the participants and researchers?	NA	Non-participants were not present.
16. Description of sample	What are the important characteristics of the sample? e.g. demographic data, date	Page 3, 4, and 5	See especially Table 1 and 2
Data collection			
17. Interview guide	Were questions, prompts, guides provided by the authors? Was it pilot tested?	Page 3, 4, and 6. Page 3 and 4 focus more about the pilot testing.	See Table 3 on page 6 for the guide.
18. Repeat interviews	Were repeat inter views carried out? If yes, how many?	Page 4	No repeat interviews.
19. Audio/visual recording	Did the research use audio or visual recording to collect the data?	Page 4 and 6	Audio-recording.
20. Field notes	Were field notes made during and/or after the interview or focus group?	Page 6	In addition to field notes, a reflexive diary and journal was kept throughout the study process.
21. Duration	What was the duration of the inter views or focus group?	Page 1, 5, and 6	
22. Data saturation	Was data saturation discussed?	Page 3 and 4	
23. Transcripts returned	Were transcripts returned to participants for comment and/or correction?	Page 4	

**Domain 3: analysis and findings**			
Data analysis			
24. Number of data coders	How many data coders coded the data?	Pages 6 and 7	
25. Description of the coding tree	Did authors provide a description of the coding tree?	Pages 6 and 7	Coding was summarized on pages 6 and 7. Table 4 in the article does illustrate the themes, subthemes, and categories. A full example of the coding tree was not included as part of the published article.
26. Derivation of themes	Were themes identified in advance or derived from the data?	Page 6 and 7	Themes were derived from the data.
27. Software	What software, if applicable, was used to manage the data?	Page 6	
28. Participant checking	Did participants provide feedback on the findings?	Page 4 and 5	
Reporting			
29. Quotations presented	Were participant quotations presented to illustrate the themes/findings? Was each quotation identified? e.g. participant number	Results section	Yes, participant quotations were presented. See results section.<br><br>Quotations were identified. However, there were just a couple of quotations for which the participant pseudonym purposefully was not given due to the more personal or sensitive nature of the quotation.
30. Data and findings consistent	Was there consistency between the data presented and the findings?	Results section	Yes. See results section.
31. Clarity of major themes	Were major themes clearly presented in the findings?	Results section	Yes. See results section
32. Clarity of minor themes	Is there a description of diverse cases or discussion of minor themes?	Results and Discussion sections	Yes. See results and discussion sections.
